# Extracellular vesicles of carcinoma-associated fibroblasts creates a pre-metastatic niche in the lung through activating fibroblasts

**DOI:** 10.1186/s12943-019-1101-4

**Published:** 2019-12-03

**Authors:** Jing Kong, Hongzhu Tian, Fuyin Zhang, Zebing Zhang, Jiao Li, Xue Liu, Xiancheng Li, Jing Liu, Xiaojie Li, Dong Jin, Xuesong Yang, Bo Sun, Tao Guo, Yong Luo, Yao Lu, Bingcheng Lin, Tingjiao Liu

**Affiliations:** 1 Department of Oral Pathology, College of Stomatology, Dalian Medical University, West Section No. 9, South Road of Lvshun, Dalian, 116044 China; 2 Department of Oral Surgery, the Second Affiliated Hospital, Dalian Medical University, Dalian, China; 3 Department of Oral Pathology, College of Stomatology, Jilin University, Changchun, China; 4 Department of Urology, the Second Affiliated Hospital, Dalian Medical University, Dalian, China; 5 Sino-UK Regenerative Medicine Center, the First Affiliated Hospital, Dalian Medical University, Dalian, China; 6 Department of Biochemistry and Molecular Biology, Liaoning Provincial Core Lab of Glycobiology and Glycoengineering, Dalian Medical University, Dalian, China; 7 Department of Thoracic Surgery, The First Affiliated Hospital, Dalian Medical University, Dalian, China; 8 Faculty of Chemical, Environmental and Biological Science and Technology, Dalian Technology University, Dalian, China; 9 Department of Biotechnology, Dalian Institute of Chemical Physics, Chinese Academy of Sciences, Dalian, China

**Keywords:** Carcinoma-associated fibroblast, Extracellular vesicles, Pre-metastatic niche, Lung metastasis

## Abstract

**Objectives:**

Carcinoma-associated fibroblasts (CAFs) have been known to promote cancer progression by modifying the primary tumor microenvironment. We aimed to elucidate the intercellular communication between CAFs and secondary organs in salivary adenoid cystic carcinoma (SACC) metastasis.

**Methods:**

Pre-metastatic and metastatic animal models of SACC were established using extracellular vesicles (EVs) from CAFs and SACC cells. Lung fibroblasts (LFs) were treated with EVs and their transcriptomic alterations were identified by RNA sequencing. ITRAQ were performed to analyze EV cargos. TC I-15 was used to inhibit EV uptake by LFs and SACC lung metastasis in vivo.

**Results:**

Here, we show that CAF EVs induced lung pre-metastatic niche formation in mice and consequently increased SACC lung metastasis. The pre-metastatic niche induced by CAF EVs was different from that induced by SACC EVs. CAF EVs presented a great ability for matrix remodeling and periostin is a potential biomarker characterizing the CAF EV-induced pre-metastatic niche. We found that lung fibroblast activation promoted by CAF EVs was a critical event at the pre-metastatic niche. Integrin α2β1 mediated CAF EV uptake by lung fibroblasts, and its blockage by TC I-15 prevented lung pre-metastatic niche formation and subsequent metastasis. Plasma EV integrin β1 was considerably upregulated in the mice bearing xenografts with high risk of lung metastasis.

**Conclusions:**

We demonstrated that CAF EVs participated in the pre-metastatic niche formation in the lung. Plasma EV integrin β1 might be a promising biomarker to predict SACC metastasis at an early stage. An integrated strategy targeting both tumor and stromal cells is necessary to prevent SACC metastasis.

## Introduction

Carcinoma-associated fibroblasts (CAFs) are considered to be activated fibroblasts in the tumor stroma and contribute to malignant initiation and progression [1, 2]. Numerous experimental and clinical studies support that CAFs secrete growth factors, chemokines, matrix metalloproteinases (MMPs) and extracellular matrix (ECM) to regulate tumor growth, angiogenesis and the recruitment of bone marrow-derived cells (BMDCs) in primary tumors, thereby promoting metastasis [3–7]. However, it remains to be determined whether CAFs could promote metastasis by modifying the microenvironment in distant organs.

It has been demonstrated that primary tumor cells are able to influence and alter the microenvironment of secondary organs by promoting the formation of supportive metastatic microenvironments, termed the pre-metastatic niche, prior to tumor cell dissemination [8–18]. Pre-metastatic niche formation is a spatio-temporal process that favors tumor cell colonization. At the initial stage of pre-metastatic niche formation, tumor-derived factors are transported by the blood flow and increase vascular permeability in the target organs [11]. Furthermore, these factors mobilize and recruit BMDCs to pre-metastatic sites through the upregulation of proinflammatory molecules [9, 10, 14]. ECM remodeling is a critical step during pre-metastatic niche formation. The deposition of fibronectin (FN), MMPs, lysyl oxidase (LOX) and other factors remodeled the tissue microenvironment to facilitate tumor cell adhesion and colonization [9, 13, 15, 16]. However, limited information is available about the role of CAFs in pre-metastatic niche formation.

Recent discoveries indicate that tumor products can be delivered to distant organs via extracellular vesicles (EVs) [19, 20]. EVs are a heterogeneous group of cell-derived membranous structures comprising small exosomes and large microvesicles [21]. EVs contain functional molecules, including proteins, mRNAs, miRNAs, lncRNAs and dsDNAs [22–27]. These cargos are protected by EVs during the delivery process, which is critical in the cellular communications between organs separated over a long distance. EVs are not only present in biological fluids, but also bind to matrices as an integral and functional component of ECM bioscaffolds [28]. Tumor cell-derived EVs have been demonstrated to participate in pre-metastatic niche formation by delivering proteins, mRNAs and microRNAs to the lung [29–31], liver [32, 33] and bone marrow [34].

In this study, we aimed to elucidate the role of CAF EVs in pre-metastatic niche formation in the lungs. Salivary adenoid cystic carcinoma (SACC) is one of the most common carcinomas in the salivary glands. It tends to metastasize in the early stages and the lungs are the most frequent metastatic site, constituting 74.5% of all metastases [35]. Thus, SACC is an ideal tumor model to study lung metastasis. We compared the roles of SACC EVs and CAF EVs in pre-metastatic niche formation. Our results indicated that CAF EVs presented a great ability for ECM remodeling by activating the TGF-β signaling pathway in lung fibroblasts (LFs). CAF EVs mainly targeted LFs via integrin α2β1 and consequently promoted their tumor permissive abilities. We also demonstrated that plasma EV integrin β1 might predict SACC lung metastasis at an early stage.

## Methods

### Cell culture

Two primary CAFs (CAF-A1 and CAF-A2) isolated from human SACC tumor tissues were used in this study. The characteristics of CAF-A1 and CAF-A2 were described previously [36]. Primary mouse LFs were isolated from the lung tissues of C5BL/6 J mice. Normal fibroblasts (NFs) were isolated from human gingival tissues excised during tooth extraction. CAFs, LFs and NFs were cultured in DMEM-F12 medium supplemented with 10% fetal bovine serum (FBS, ScienCell, California, USA), 10 ng/mL bFGF (PeproTech, New Jersey, USA), 10 ng/mL EGF (PeproTech), 100 U/mL penicillin and 100 U/mL streptomycin (ThermoFisher, Massachusetts, USA) at 37 °C with 5% CO_2_. A SACC cell line (SACC-LM) with enhanced lung metastatic behavior was used in this study. It was a kind gift from Prof. Shenglin Zhang, Beijing University and cultured in DMEM-F12 medium supplemented with 10% FBS, 100 U/mL penicillin and 100 U/mL streptomycin at 37 °C with 5% CO_2_.

### Human tissue samples

All human SACC tissues used in this study were collected from patients in the School of Stomatology, Jilin University. A total of 15 SACC tissues (paraffin-embedded tissues) were collected from patients undergoing surgical resection. Among 15 cases of SACC, five cases had lung metastases. Immunohistochemical staining was performed to detect the expression of FAP using the streptavidin–biotin complex technique (Additional file 1: Supplementary methods).

### EV isolation and labeling

FBS was ultracentrifuged at 100,000 *g* for 70 min to remove bovine EVs. Cells were cultured for 3 days in media supplemented with 0.5% EV-depleted FBS for EV isolation. Then the cell culture medium was sequentially centrifuged at 500 *g*, 2500 *g* and 12,000 *g* and the supernatant was collected. After ultracentrifugation at 100,000 *g* for 70 min, pellet was isolated and resuspended in 20 mL PBS. Then EVs were isolated from PBS solution using Total Exosome Isolation Reagent (Invitrogen 4,478,359).

Plasma EVs of mice were isolated. Blood was collected into an EDTA-K2 anticoagulant tube and mixed immediately to avoid clotting. Blood was sequentially centrifuged at 1500 *g* and 2400 *g* for 10 min at 4 °C, the supernatant was collected and diluted at ratio 1:1 with PBS. Then EVs were isolated using Total Exosome Isolation Reagent according to the manufacturer’s instruction.

Purified EVs were labeled with PKH67 membrane dye (Sigma-Aldrich) according to the manufacturer’s protocol. Briefly, EVs (50 μg) were suspended in 1 mL PBS before 1 mL Diluent C was added. Meanwhile, 4 μL PKH67 was added to 1 mL Diluent C and mixed gently with the EV solution for 4 min. Then 2 mL of 1% BSA/PBS was added to bind excess dye. Fluorescently-labeled EVs were then washed with PBS and re-extracted with Total Exosome Isolation Reagent.

### Pre-metastatic niche study

EVs (5 μg/ mouse/ treatment in 50 μL PBS) were injected into C57BL/6 J mice via the tail vein every other day for 3, 7 or 14 days. As a control, mice were injected with the same volume of PBS. At different time points, mice were euthanized and the lung tissues were collected, fixed with 4% paraformaldehyde and 30% sucrose solution overnight, and embedded into Tissue-Tek® O.C.T. Compound (OCT, SAKURA, California, USA).

Another pre-metastatic model was established using BALB/c nude mice aged 3–4 weeks old (about 18 g, female, Dalian Medical University Laboratory Animal Center). SACC-LM cells were injected with or without CAFs into subcutaneous space of the flank. Mice were divided into four groups according to the types of transplanted tumor cells: the SACC-LM group (2.25 × 10^6^ cells/mouse), the SACC-LM + CAF-A1 group (2 × 10^6^ SACC-LM + 0.25 × 10^6^ CAF-A1/mouse), SACC-LM + CAF-A2 group (2 × 10^6^ SACC-LM + 0.25 × 10^6^ CAF-A2/mouse) and the control group (without cell transplantation), with five mice in each group. The nude mice were raised in sterile conditions for 3 weeks, then euthanized. The lung tissues were removed and fixed with 4% paraformaldehyde and 30% sucrose solution overnight, then embedded into OCT. Whole blood of each mice was collected into an EDTA-K2 anticoagulant tube for plasma EV isolation.

Tissues embedded in OCT were sectioned into 8 μm thick slices. The sections were blocked with 10% goat serum or 3% BSA and stained with FN (1:200, Abcam, Cambridgeshire, UK), LOX (1:300, Abcam), MMP9 (1:500, Abcam), periostin (POSTN, 1:500, Abcam) and vascular endothelial growth factor receptor-1 (VEGFR1, 1:250, Abcam). Secondary antibodies conjugated to Alexa Fluor 488 or 594 (1:200, Abbkine, California, USA) were used to detect different primary antibodies. Nuclei were counterstained with diamidino-2-phenylindole (DAPI, 1:4000, ThermoFisher). Sections were mounted with fluorescence mounting media (VECTOR, California, USA) and visualized using an inverted microscope (Olympus IX71, Tokyo, Japan). The expression area of FN, LOX, MMP9, and POSTN and the number of VEGFR1 positive cells were analyzed using Image-Pro® Plus version 6.0 software.

### Metastasis study

To analyze the role of EVs in SACC-LM metastasis, C57BL/6 J mice aged 8 weeks old were used (about 18 g, female, Dalian Medical University Laboratory Animal Center). Mice were divided into four groups according to the types of pre-treated EVs: the SACC-LM EV group, the CAF-A1 EV group, the CAF-A2 EV group and the control group, with five mice in each group. EVs (5 μg/ treatment in 50 μL PBS) were injected into the tail vein every other day for 3 weeks. Then, SACC-LM cells (2.5 × 10^5^ cells/mouse in 100 μL PBS) were injected via the tail vein. EVs were continuously injected into the tail vein twice a week. PBS injection was used as a control. Three weeks after SACC-LM injection, mice were euthanized and the lungs was fixed and embedded into OCT.

To investigate the role of CAFs in SACC lung metastasis and survival, subcutaneous xenografts using nude mice were performed. The transplanted cell type and number were the same as that of the pre-metastatic niche model using nude mice. Two sets of experiment were conducted. In the first set, the nude mice were raised in sterile conditions for 5 weeks. Both xenografts and lung tissues were harvested and fixed with 4% paraformaldehyde and 30% sucrose solution overnight, then embedded into OCT. Meanwhile whole blood of each mice was collected for plasma EV isolation. In the second set of experiment, mice were raised until they died naturally and survival was analyzed.

Sections of xenografts and the lungs with 8 μm-thickness were prepared and stained with hematoxylin and eosin (HE). The presence of CAF-A1/A2 in the xenografts was confirmed by immunofluorescent staining with anti-human fibroblast activation protein (FAP) antibody (1:250, Proteintech). Microvessel density (MVD) of subcutaneous xenografts was evaluated by counting CD34-labeled vessels (Additional file 1: Supplementary methods). Micrometastasis in the lung tissues were confirmed by immunofluorescent staining with anti-human Pan CK antibody (Merck Millipore, California, USA). Metastatic colony number and area were used to evaluate the metastasis. Images were recorded by an inverted microscope and calculated using Image-Pro® Plus version 6.0 software.

### LFs treatment with EVs

To visualize EV uptake by primary mouse LFs, cells were seeded into a 48-well plate with 3000 cells/well in triplicate. EVs were suspended in culture medium and added into cell culture wells (50 μg/well). After 4 h incubation at 37 °C, the cell culture medium was removed and cells were rinsed with PBS. Images were recorded by an inverted fluorescent microscope. Wound healing assay was performed to evaluate cell migration ability (Additional file 1: Supplementary methods). To evaluate the activation of mouse LFs by EVs, cells were seeded in a 24-well plate in triplicate (1 × 10^5^ cells/well). Then EVs in culture medium were added (25 μg/well) and incubated for 3 days. The cell culture medium containing EVs was changed every other day. After 3 days, cells were fixed with 4% paraformaldehyde, washed with PBS, and stained with FAP, (1:200, Abcam), α-SMA (1:200, Proteintech), POSTN (1:500, Abcam), Ki-67 (1:100, Proteintech), and p-Smad 3 (1:1000, Abcam). Immunoreactions were detected with FITC-labeled goat anti-rabbit secondary antibody (1:200, Abbkine). Nuclei were counterstained with DAPI. For nuclear proteins (Ki67 and p-Smad3), the number of positive cells were calculated. For cytoplasmic proteins (FAP, α-SMA and POSTN), the fluorescent signal was measured with Image-Pro® Plus version 6.0.

### TC I-15 inhibition assay in vitro and in vivo

To inhibit EV uptake by LFs in vitro, cells were seeded into a 48-well plate with 3000 cells/well in triplicate. Then PKH67-labeled EVs (50 μg/well) were incubated with 0, 0.5, 1 and 2 μM TC I-15 (TOCRIS, Oxfordshire, UK) at 37 °C for 1 h. Then EVs with TC I-15 were added to the LF culture wells and incubated at 37 °C for 4 h. After washing with PBS, cells were imaged under an inverted fluorescent microscope.

To inhibit LF activation induced by EVs in vitro, cells were seeded into a 24-well plate with 1 × 10^5^ cells/well in triplicate. EVs (25 μg/well) were incubated with 2 μM TC I-15 in culture medium at 37 °C for 1 h, then added into LF culture wells and incubated for 3 days. The cell culture medium containing EVs and TC I-15 was changed every other day. After 3 days, cells were fixed with 4% paraformaldehyde, washed with PBS, and stained with FAP (1:200, Abcam), α-SMA (1:200, Proteintech), POSTN (1:500, Abcam), Ki-67 (1:100, Proteintech), and p-Smad 3 (1:1000, Abcam). Immunoreactions were detected with FITC-labeled goat anti-rabbit secondary antibody (1:200, Abbkine). Nuclei were counterstained with DAPI.

To investigate whether TC I-15 could suppress EV-induced pre-metastatic niche formation in vivo, C57BL/6 J mice were used. EVs (5 μg) were incubated with TC I-15 (10 μg) in 50 μL PBS at 37 °C for 1 h. Then EVs and TC I-15 were injected into mice via the tail vein every other day. After 3 days, mice were euthanized and the whole lung tissues were collected to examine ECM protein expression.

To investigate whether TC I-15 could suppress EV-induced SACC lung metastasis, EVs (5 μg) were pre-incubated with TC I-15 (10 μg) in 50 μL PBS and injected into C57BL/6 J mice via tail vein every other day for 3 weeks. Then, SACC-LM cells (2.5 × 10^5^ cells/mouse in 100 μL PBS) were injected via the tail vein. EVs and TC I-15 were continuously injected into the tail vein twice a week. PBS was used as a control. Three weeks after SACC-LM injection, mice were euthanized and the lungs were fixed and embedded into OCT.

### Statistical analyses

Statistical analyses were performed using SPSS version 13.0 for Windows. Each experiment was conducted at least three times. The Mann–Whitney *U* text was conducted for comparisons between all of the data. Data were expressed as the mean ± SD. A *p* value of < 0.05 was considered statistically significant.

## Results

### CAF EVs promoted SACC lung metastasis

**Fig. 1:**
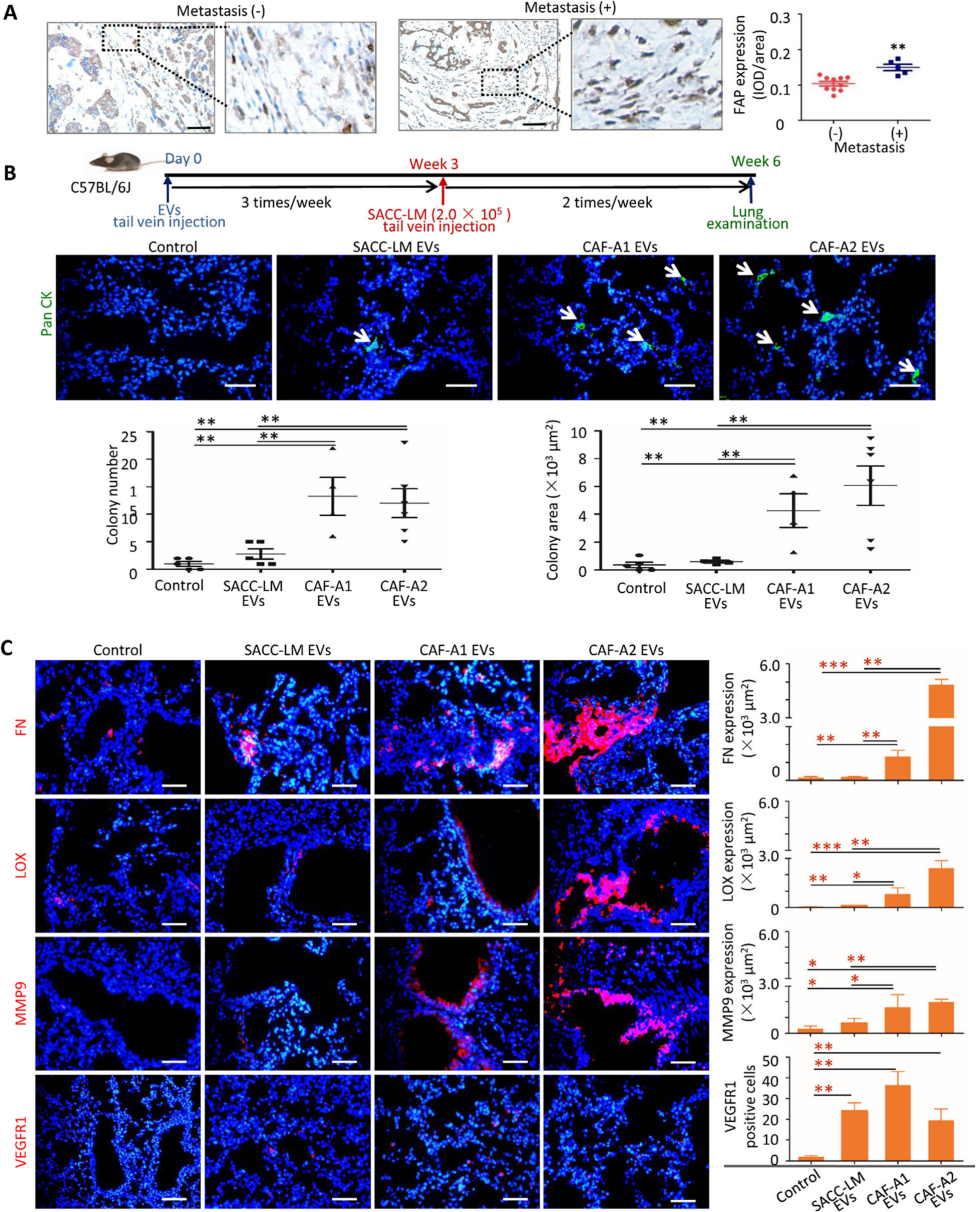
CAF EVs promoted SACC lung metastasis and pre-metastatic niche formation. **a** FAP expression and quantification in the stroma of human SACC with or without lung metastasis. Scale bar = 50 μm. **b** Metastatic SACC-LM cells were detected by immunofluorescent staining with anti-human Pan CK antibody. C57BL/6 J mice were pre-treated with SACC-LM EVs (*n* = 5), CAF-A1 EVs (*n* = 4) and CAF-A2 EVs (*n* = 5). Scale bar = 50 μm. **c** Immunofluorescence staining and quantification of FN, LOX and MMP9 in lung sections from tumor-free mice injected every other day for 3 days with the indicated EVs (*n* = 5 per group). Immunofluorescence staining and quantification of VEGFR1+ cells in lung sections from tumor-free mice injected every other day for 14 days with the indicated EVs (*n* = 5 per group). Scale bar = 50 μm. * *P* < 0.05, **

Lung is the most common metastatic site of human SACC. To investigate whether CAFs in SACC stroma correlated with lung metastasis, 15 cases of SACC were collected, including 5 cases with lung metastasis and 10 without lung metastasis. Immunohistochemical staining identified higher expression of FAP, a biomarker of CAFs, in the stroma of SACC cases with lung metastasis than those without lung metastasis **(**Fig. 1a**)**, suggesting that CAFs may contribute to SACC lung metastasis.

As EVs play important roles in the communication between primary tumor and distant organs, we analyzed whether CAF EVs could promote SACC metastasis. EVs from SACC-LM, CAF-A1 and CAF-A2 cells were isolated and the presence of CD63, CD81, CD9, and HSP-70 and absence of CALNEXIN were confirmed by western blot analysis (Additional file 2: Figure S1A). Their morphologies were confirmed by transmission electron microscopy (TEM) (Additional file 2: Figure S1B). EV size distribution and particle concentration were quantified by a nanoparticle tracing system (Additional file 2: Figure S1C). The particle number per μg of protein was analyzed (Additional file 2: Figure. S1D).

To elucidate EV distribution in vivo, the same amount of EVs from SACC-LM, CAF-A1 and CAF-A2 cells labeled with PKH67 were injected into the retro-orbital sinus and tail vein of C57BL/6 J mice, respectively. After 24 h, the common SACC metastatic organs, including the lungs, liver and brain, were examined. Regardless of the route of injection, the three types of EVs were observed principally in the lungs, rarely in the liver, and not in the brain (Additional file 2: Figure S2), suggesting that EVs from both SACC cells and SACC-derived CAFs showed prominent lung tropism.

Then we examined whether these EVs could promote SACC lung metastasis. Three types of EVs were injected into C57BL/6 J mice via the tail vein three times a week for 3 weeks, respectively. SACC-LM cells were then injected into these pre-treated mice via the tail vein. PBS was used as a control. At week 6, lung tissues were harvested and examined. Metastatic colonies were found in the lungs of mice with EV pre-treatment, but rarely in the control **(**Fig. 1b**)**. Both metastatic colony numbers and area were significantly increased in the CAF-A1/A2 EV groups compared with those in the SACC-LM EV group and control. These results suggested that CAF EVs presented a greater ability to promote SACC lung metastasis than SACC-LM EVs.

### CAF EVs presented a greater ECM remodeling ability than SACC EVs at the pre-metastatic niche

As pre-treatment of mice with EVs from CAF-A1 and CAF-A2 increased SACC lung metastasis, we hypothesized that these EVs may modify the lung microenvironment to facilitate tumor cell colonization. To evaluate the microenvironment changes in the lungs, mice were pre-treated with SACC-LM EVs, CAF-A1 EVs or CAF-A2 EVs for 3, 7 and 14 days. The presence of known markers for the pre-metastatic niche, including FN, LOX, and MMP9, was examined by immunofluorescent staining. For all three markers, higher expression was detected in the CAF-A1/A2 EV groups than in the SACC-LM EV group and controls as early as day 3 **(**Fig. 1c**)**. By contrast, FN, LOX and MMP9 showed similar expression in the SACC-LM EV group and controls at day 3 and significantly higher expression in the SACC-LM EV group than in controls at day 7 (Additional file 2: Figure. S3A). These data indicated that the expression of biomarkers related to pre-metastatic niche formation was earlier in the CAF EV group than in the SACC EV group. Particularly, LOX sustained higher expression in the CAF-A1 EV group than in the SACC EV group until day 14, and FN sustained higher expression in the CAF-A2 EV group than in the SACC EV group (Additional file 2: Figure. S3A–B). These data suggested that CAF EVs possess a greater ECM-remodeling ability than SACC EVs.

BMDC recruitment is an important event in pre-metastatic niche formation. We examined the quantity of VEGFR1-positive cells in the lungs of mice with EV pre-treatment compared with the controls. It was found that EV treatment increased the number of VEGFR1-positive cells significantly in the lungs, compared with the controls **(**Fig. 1c**)**. No significant difference was found between the SACC EV group and the CAF-A1/A2 EV groups. These data suggested that CAF EVs could recruit BMDCs to the lungs, but they did not display superiority to SACC EVs in this respect.

### CAF EVs activated LFs and improved their tumor-permissive abilities

**Fig. 2:**
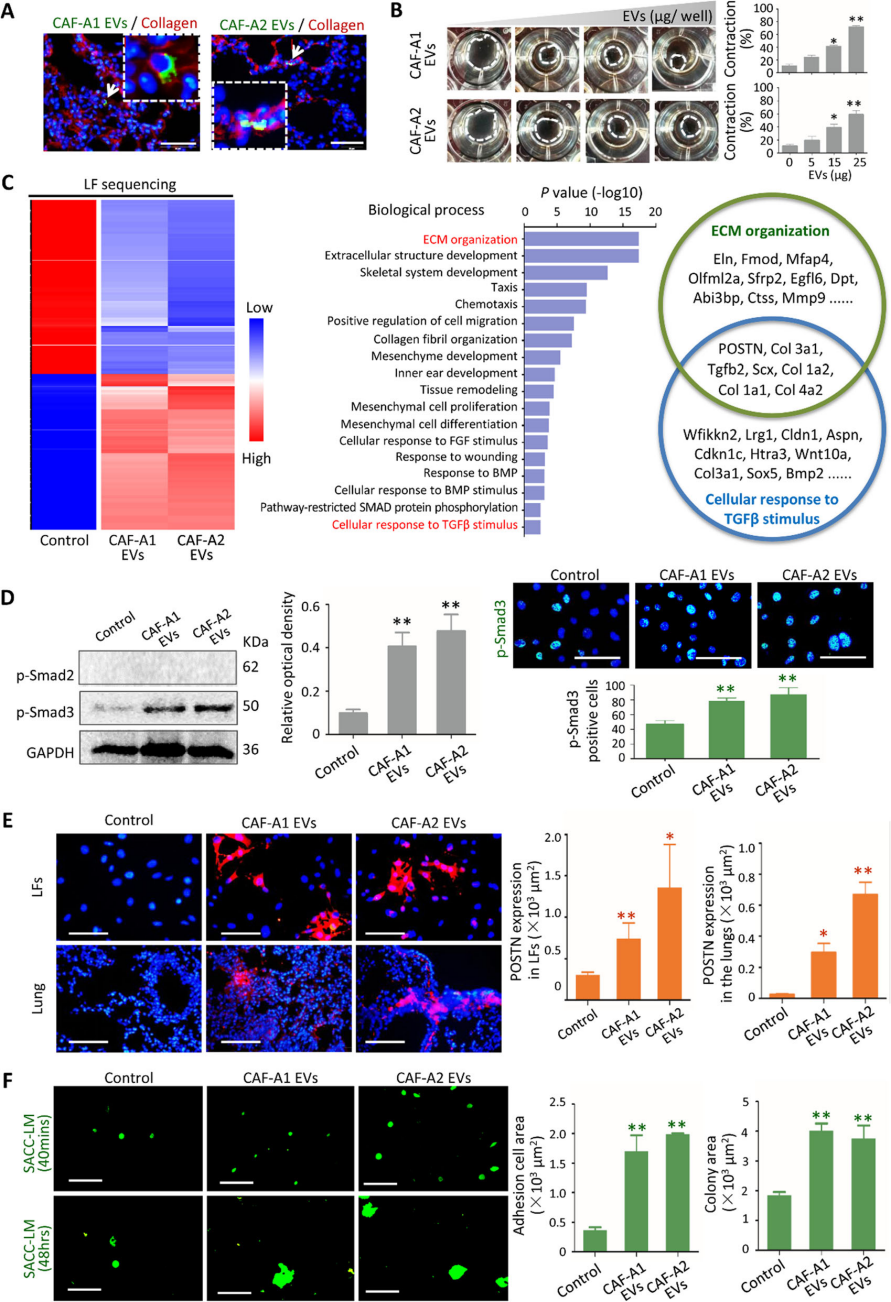
CAF EVs activated LFs via TGF-β signaling pathway. **a** CAF-A1/A2 EVs were labeled with PKH67 (green) and the lung tissue sections were stained with Collagen I antibody (red). Scale bar = 50 μm. **b** Collagen gel contract assay using EVs from CAF-A1 and CAF-A2. EV concentrations tested were 0, 5, 15, 25 μg/well. **c** Heatmap of RNA sequencing. Enriched Biological Processes with GO term using differentially expressed genes were listed. POSTN was the top one among the enriched gene in both *ECM Organization* and *Cellular Response to TGF-β Stimulus* Biological Processes. **d** Expression of p-Smad2 and p-Smad3 in LFs with or without CAF EV pre-treatment assessed by western blot and immunofluorescent staining. Scale bar = 100 μm. **e** POSTN expression in LFs in vitro and in the lung tissues of mice (*n* = 5 per group) treated with CAF-A1/A2 EVs for 3 days. Scale bar = 100 μm. **f** Images and quantization of adhesion and proliferation of SACC-LM-GFP cells (green) on LFs treated with EVs from SACC-LM, CAF-A1 and CAF-A2. Scale bar = 100 μm. * *P* < 0.05, ** *P* < 0.01, *** *P* < 0.001

To identify the cells able to uptake EVs in the lungs, PKH67-labeled EVs from SACC-LM, CAF-A1 and CAF-A2 cells were injected into C57BL/6 J mice. After 24 h, lung tissues were collected and stained with antibodies against collagen I, fibroblast-specific protein 1 (FSP1), CD34 and EpCAM. CAF-A1 and CAF-A2 EVs co-localized with Collagen I- and FSP1-positive cells in the lungs, but not CD34- and EpCAM-positive cells (Fig. 2a **and** Additional file 2: Figure S4A). By contrast, co-localization of SACC-LM EVs with CD34-positive cells could be observed (Additional file 2: Figure. S4A). To further confirm the direct target cells of CAF EVs, primary LFs of mice were isolated and incubated with PKH 67-labeled EVs in vitro. As shown in Additional file 2: Figure. S4B, large numbers of CAF-A1/A2 EVs were taken up by LFs, whereas only small numbers of SACC-LM EVs were internalized by LFs. This suggested that LFs were the major target cells of CAF EVs but not of SACC-LM EVs. We then investigated the functional consequences of CAF EV uptake by LFs. We found that CAF EVs increased the migration ability of LFs and the expression of FAP, α-SMA and Ki67 in LFs, compared with SACC-LM EVs and controls (Additional file 2: Figure. S5A-C). As shown in Fig. 2b**,** CAF-A1/A2 EVs promoted collagen gel contraction in a dose-dependent manner. These data suggest that LFs were activated by CAF EVs and presented the phenotypes of activated fibroblasts. Additionally, we treated LFs with NF EVs (Additional file 2: Figure. S6A-C). LFs rarely internalized NF EVs. Consequently, the expression of FAP, α-SMA, Ki67, and POSTN in LFs and collagen gel contract didn’t change by NF EV treatment.

To reveal the mechanisms by which LFs were activated by CAF EVs, we pre-treated mouse LFs with CAF-A1/A2 EVs in vitro and performed gene expression analysis by RNA sequencing **(**Fig. 2c**)**. Gene Ontology (GO) analysis revealed that differentially expressed mRNAs were significantly enriched in the Biological Processes of *ECM Organization*, *Extracellular Structure Development, Skeletal system development, Cellular Response to TGF-β Stimulus* etc. POSTN was the top one among the upregulated genes enriched in the Biological Processes of *ECM Organization* and *Cellular Response to TGF-β Stimulus*. The enriched Cellular Component and Molecular Function by GO analyses were shown in Additional file 3: Tables S1 and S2.

TGF-β signaling pathway has been reported to be one of the major pathways by which fibroblasts are activated with increased expression of FAP and α-SMA. Then we investigated whether TGF-β signaling pathway was activated in LFs pre-treated with CAF EVs. Western blot confirmed the expression of phosphorylated Smad3 (p-Smad3) in LFs by CAF EV treatment **(**Fig. 2d**)**. Cell immunofluorescent staining revealed p-Smad3 expressed in the nuclei and positive cell number significantly increased with CAF-A1/A2 EV treatment, compared with the control **(**Fig. 2d**)**. It suggested that TGF-β signaling pathway participated LF activation induced by CAF EVs. By contrast, NF EVs failed to induce p-Smad3 expression in LFs (Additional file 2: Figure. S6D).

Next, we examined whether CAF EVs could induce POSTN expression in LFs. Immunofluorescent staining confirmed POSTN expression in LFs pre-treated with CAF-A1/A2 EVs, compared with the control **(**Fig. 2e**)**. Then we investigated whether POSTN was a biomarker of the pre-metastatic niche created by CAF EVs in vivo. It was demonstrated that POSTN expression increased significantly in the lungs of mice with CAF-A1/A2 EV treatment as early as day 3 **(**Fig. 2e**)**. By contrast, POSTN expression did not changed significantly in the lungs of mice with SACC-LM EV treatment at day 3 (data not shown). It suggested that POSTN might be a potential biomarker characterizing the CAF EV-induced pre-metastatic niche in the lungs at an early time.

To evaluate the tumor permissive ability of LFs treated with CAF EVs, SACC-LM-GFP cells were added to the cell culture wells containing LFs pre-treated with CAF-A1/A2 EVs **(**Fig. 2f**)**. The results indicated that more tumor cells adhered to LFs treated with CAF-A1/A2 EVs, compared with the controls. When these adhesive tumor cells were cultured with the indicated EVs for another 48 h, tumor colonies formed. The tumor colony area was significantly larger in the CAF-A1/A2 EV groups than in the control group. Taken together, these findings indicated that CAF EVs improved the tumor permissive abilities of LFs, in terms of tumor cell adhesion and colony formation.

### CAF EVs contained distinct protein cargos relating to TGF-β signaling pathway

**Fig. 3:**
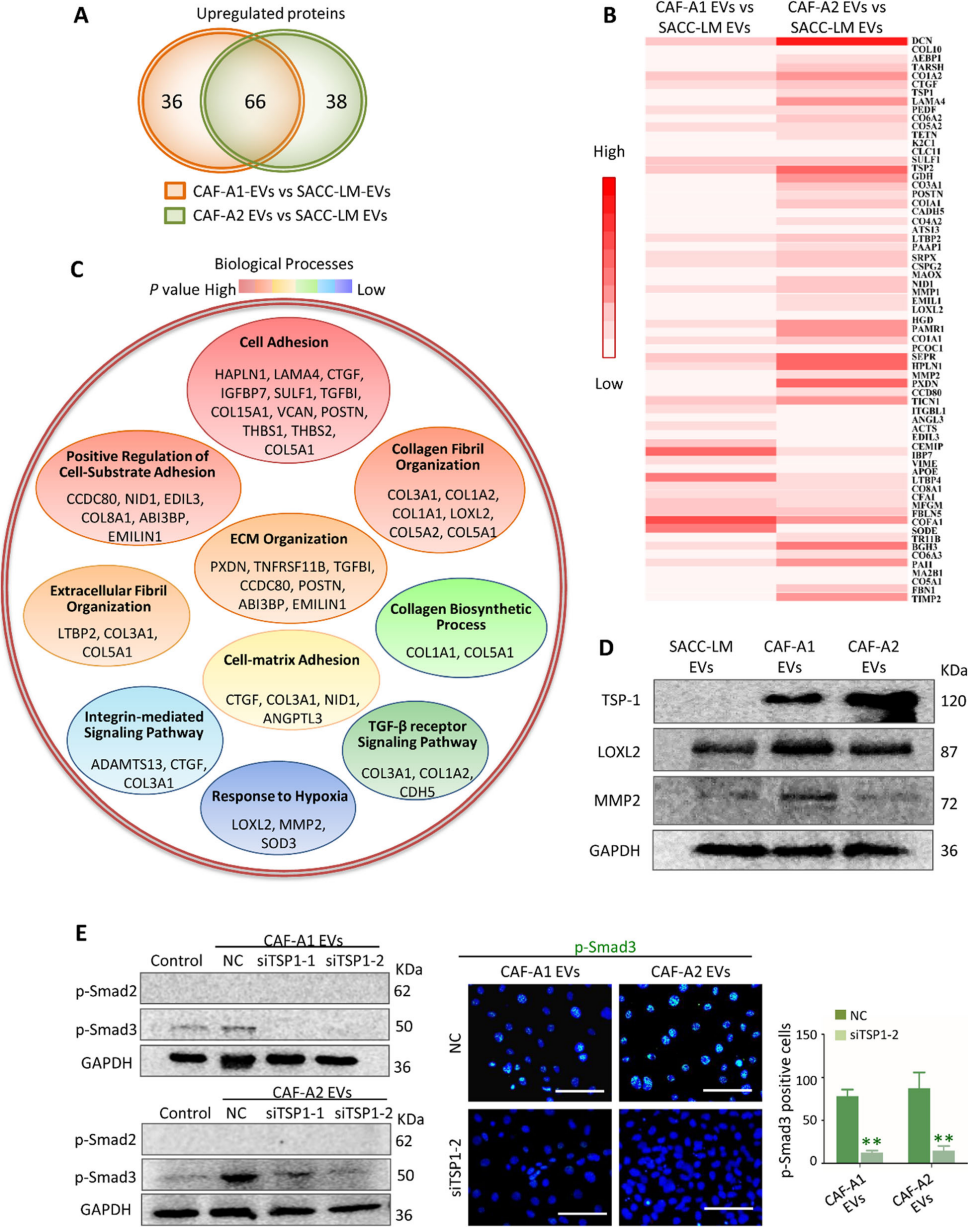
Comparison of proteins between CAF-A1/A2 EVs and SACC-LM EVs by ITRAQ. **a** Overexpressed proteins in the two comparison groups (CAF-A1 EVs vs. SACC-LM EVs, CAF-A2 EVs vs. SACC-LM EVs). **b** Heat map of the 66 proteins overexpressed in both CAF-A1 and CAF-A2 EVs, compared with SACC-LM EVs. **c** Biological Process enrichment of the 66 overexpressed proteins. **d** TSP1, LOXL2, and MMP2 expression in the EVs from SACC-LM, CAF-A1 and CAF-A2 cells by western blot. **e** TSP1 was downregulated in CAF-A1/A2 EVs by TSP1-specific siRNA transfection. Expression of p-Smad3 in LFs treated with CAF-A1/A2 EVs-NC, CAF-A1/A2 EVs-siTSP1–1, and CAF-A1/A2 EVs-siTSP1–2 assessed by western blot and immunofluorescent staining. Scale bar = 100 μm. ** *P* < 0.01

To elucidate the mechanism of LF activation by CAF EVs, we compared protein expression between SACC-LM EVs, CAF-A1 EVs and CAF-A2 EVs by isobaric tags for relative and absolute quantification (ITRAQ). A total of 928 proteins were identified in three types of EVs. Our data demonstrated higher expression of 102 proteins in CAF-A1 EVs than in SACC-LM EVs, higher expression of 104 proteins in CAF-A2 EVs than in SACC-LM EVs, and higher expression of 66 proteins in both CAF-A1 and CAF-A2 EVs than in SACC EVs **(**Fig. 3a**)**. Heatmap of the 66 differentially expressed genes in each group was shown in Fig. 3b. A program based on Biological Process, Cellular Component, and Molecular Function using DAVID tool was used to analyze the 66 differentially expressed proteins. In the “Biological Processes” category, proteins mainly participated in *Cell Adhesion*, *Positive Regulation of Cell-Substrate Adhesion*, *Collagen Fibril Organization*, *Extracellular Fibril Organization*, *ECM Organization*, *Extracellular Fibril Organization*, *Cell-matrix Adhesion*, *Collagen Biosynthetic Process*, *TGF-β receptor Signaling Pathway*, *Integrin-mediated Signaling Pathway*, and *Response to Hypoxia* **(**Fig. 3c**)**. In the “Cellular Component” category, proteins mainly accounted for the classification of *Extracellular space* (GO: 0005615, 34/66), *Extracellular matrix* (GO: 0031012, 18/66), *Proteinaceous extracellular matrix* (GO: 0005578, 20/66), *Collagen trimer* (GO: 0005581, 9/66), *Extracellular exosome* (GO: 0070062, 30/66) (Additional file 3: Table S3). In the “Molecular Function” category, proteins mainly accounted for *Extracellular matrix structural constituent* (GO: 0005201, 10/66), *Heparin binding* (GO: 0008201, 11/66), *Calcium ion binding* (GO: 0005509, 17/66), *Extracellular matrix binding* (GO: 0050840, 5/66), *Hyaluronic acid binding* (GO: 0005540, 3/66) (Additional file 3: Table S4).

The expression of thrombospondin-1 (TSP1), LOXL2 and MMP2 in CAF-A1/A2 EVs was confirmed by western blot **(**Fig. 3d**)**. TSP1, a major activator of TGF-β signaling, showed prominently high expression in CAF-A1/A2 EVs and rare expression in SACC-LM EVs. We observed that downregulation of TSP1 by siRNA transfection prevented p-Smad3 expression in LFs after treatment with CAF EVs **(**Fig. 3e**)**. Additionally, the absence of TGF-β protein in CAF EVs was confirmed by western blot (Additional file 2: Figure. S7A). Together these data suggest the specificity of TSP1 contribution to TGF-β pathway activation in LFs.

### Integrin α2β1 mediated CAF EV uptake by LFs

**Fig. 4:**
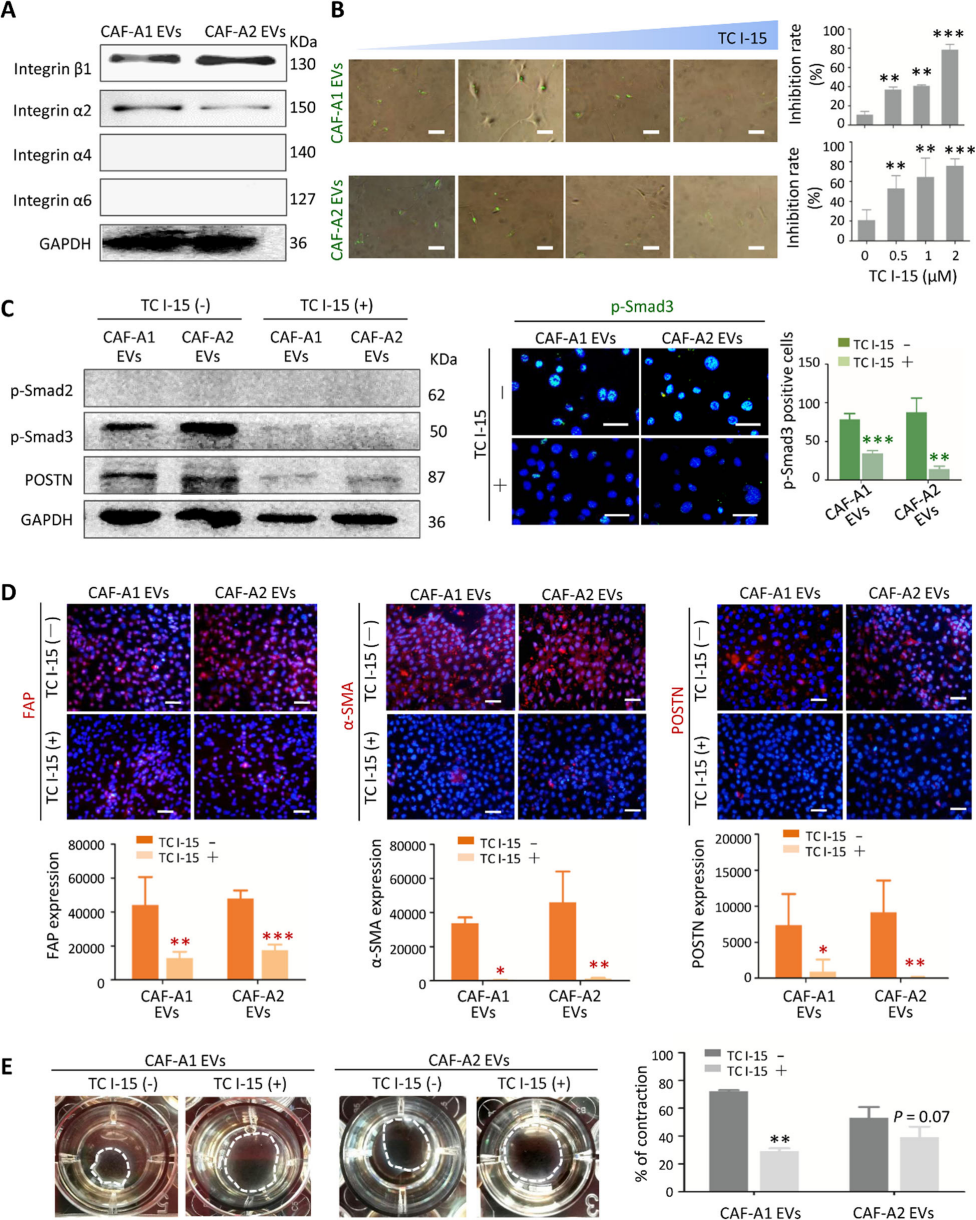
Integrin α2β1 mediated CAF EV uptake by LFs. **a** Western blot analysis of integrin expression in CAF-A1/A2 EVs. **b** Inhibition assay of CAF EV uptake by LFs in vitro*.* The TC I-15 concentrations tested were 0, 0.5, 1 and 2 μM. **c** TC I-15 inhibitory effects on the expression of p-Smad3 in LFs induced by CAF-A1/A2 EVs. **d** TC I-15 inhibitory effects on the expression of FAP, α-SMA and POSTN in LFs induced by CAF-A1/A2 EVs. **e** TC I-15 inhibitory effects on collagen gel contract promoted by CAF-A1/A2 EVs. Scale bar = 50 μm. * *P* < 0.05, ** *P* < 0.01, *** *P* < 0.001

We next investigated whether blocking of CAF EV uptake could impact LF activation. It has previously been established that unique EV integrins mediate EV uptake in specific target cells [29]. This prompted us to analyze the integrin expression in CAF EVs. We confirmed high expression of integrin β1 in CAF-A1/A2 EVs, but not in SACC-LM and NF EVs (Fig. 4a **and** Additional file 2: Figure S7A). As integrins function as heterodimers (α and β subunits), we tried to find α partners of integrin β1 in CAF EVs. Western blot analysis revealed integrin α2 expression in CAF-A1/A2 EVs, but hardly detectable integrin α4 and α6 expression **(**Fig. 4a**)**. This suggested that integrin α2β1 may mediate CAF EV uptake by LFs. Moreover, we observed that downregulation of EV integrin β1 by siRNA transfection inhibited CAF EV uptake by LFs both in vitro and in vivo, leading to downregulation of pre-metastatic biomarker expression in the lungs (Fig. S7B-G), suggesting the specificity of integrin β1 contribution to CAF EV uptake by LFs.

Then we demonstrated that TC I-15, an inhibitor of integrin α2β1 [37], suppressed CAF-A1/A2 EV uptake by LFs in a dose-dependent manner and without obvious toxic effect (Fig. 4b **and** Additional file 2: Figure. S8). Furthermore, it was found that p-Smad3 expression in LFs pre-treated with CAF-A1/A2 EVs decreased greatly with TC I-15 treatment **(**Fig. 4c**)**, suggesting that TGF-β pathway activation in LFs was suppressed by TC I-15. The expression of FAP, α-SMA and POSTN in LFs pre-treated with CAF-A1/A2 EVs greatly decreased by TC I-15 **(**Fig. 4d**)**. Collagen contract assay also demonstrated that TC I-15 attenuated the collagen gel contraction promoted by CAF-A1/A2 EVs **(**Fig. 4e**)**. These data indicated that blocking EV integrin α2β1 attenuates CAF EV uptake by LFs. Consequently, LF activation was inhibited.

### Blocking EV integrin α2β1 impacted the pre-metastatic niche formation in the lung and SACC metastasis

**Fig. 5:**
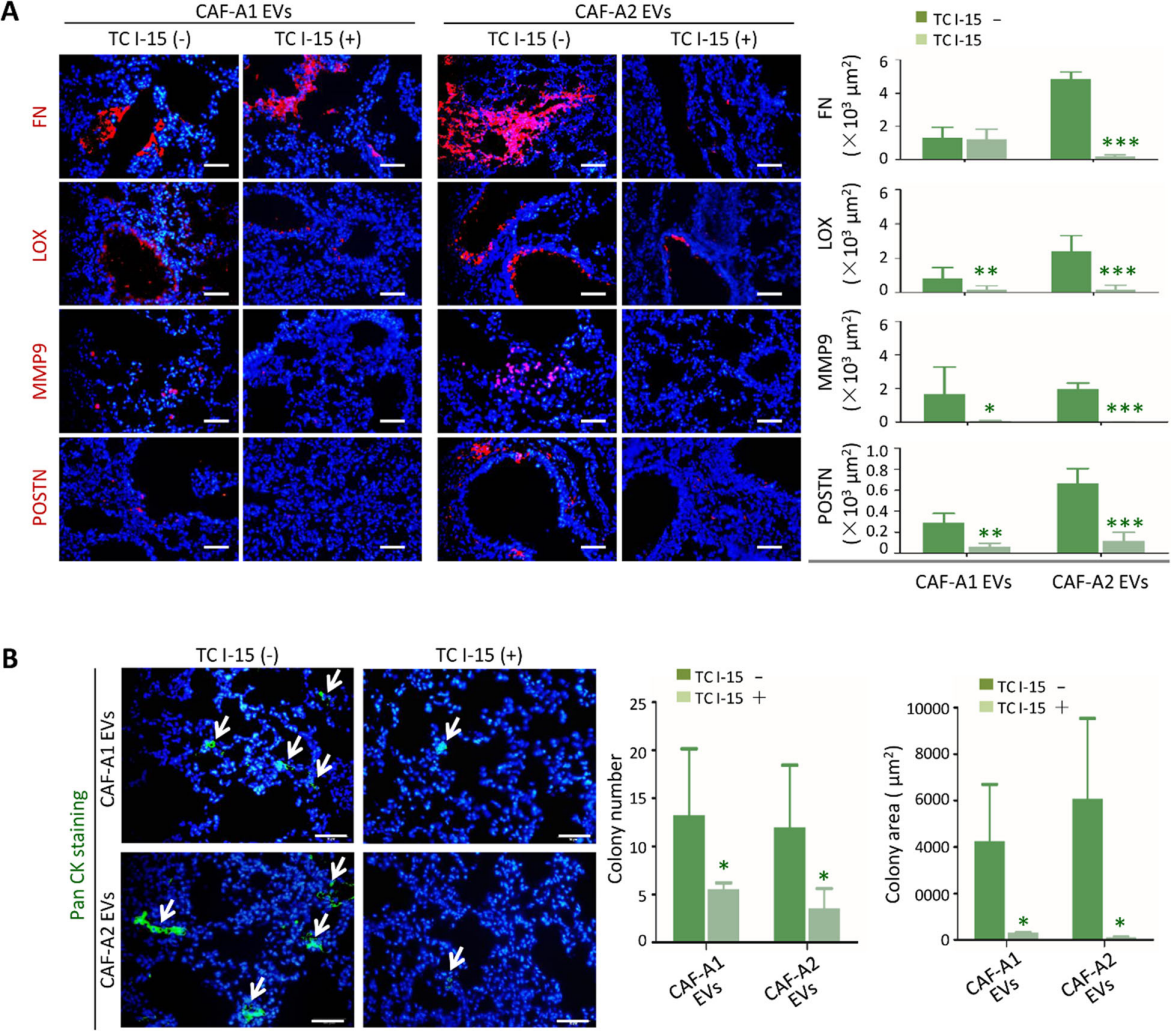
TC I-15 inhibited CAF EV-induced pre-metastatic niche formation and metastasis in vivo. **a** TC I-15 inhibitory effects on the expression of FN, LOX, MMP9, and POSTN induced by CAF-A1/A2 EVs in the lungs (*n* = 5 per group). **b** TC I-15 inhibitory effects on lung metastasis promoted by CAF-A1/A2 EVs (*n* = 5 per group). Scale bar = 50 μm. * *P* < 0.05, ** *P* < 0.01, *** *P* < 0.001

Next, we investigated whether TC I-15 could inhibit the pre-metastatic niche formation and subsequent metastasis promoted CAF EVs in vivo. TC I-15 inhibited the lung tropism of CAF-A1/A1 EVs, especially for CAF-A2 EVs (data not shown). The expression of FN, LOX, MMP9 and POSTN induced in the lungs by CAF-A1/A2 EVs was significantly decreased with TC I-15 treatment at day 3 **(**Fig. 5a**)**. At week 6, lung tissues were harvested and examined. Both metastatic colony numbers and area were significantly decreased in the TC I-15 treatment groups, compared with the groups without TC I-15 treatment **(**Fig. 5b**).**

### Integrin β1-positive plasma EVs correlated with SACC lung metastasis

**Fig. 6:**
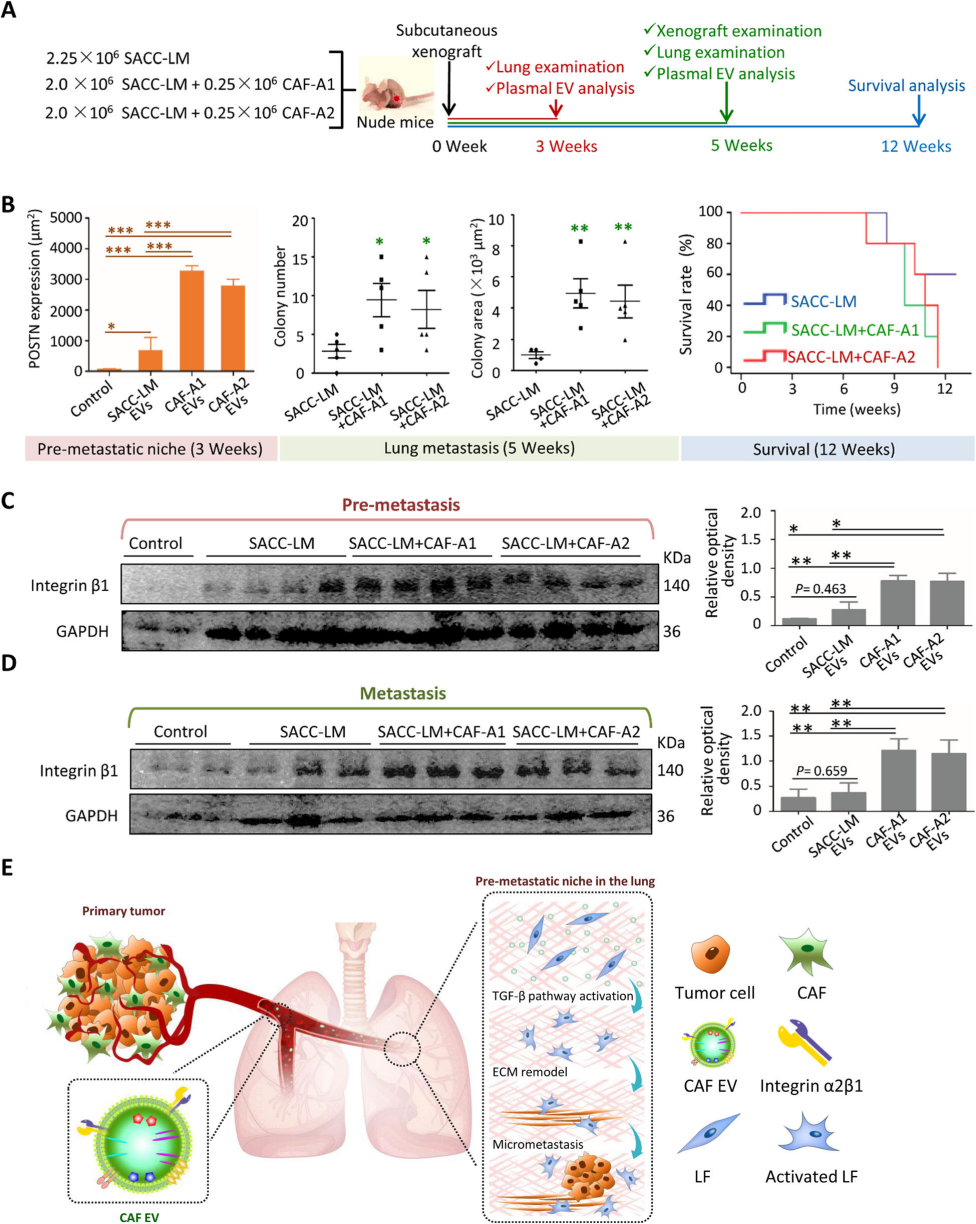
Plasma EV integrin β1 predicted SACC lung metastasis in mice. **a** Schematic of the animal experiments using BALB/c nude mice. **b** POSTN expression in the lungs of mice at week 3. Metastatic colony number and area in the lungs of mice at week 5. Survival analysis for the mice raised for up to 12 weeks. **c** Western blot analysis of plasma EV integrin β1 expression in the mice at the pre-metastatic stage. **d** Western blot analysis of plasma EV integrin β1 expression in the mice at the metastatic stage. **e** Illustration of the CAF EVs constructed pre-metastatic niche in the lungs. Scale bar = 50 μm. * *P* < 0.05, ** *P* < 0.01

To further evaluate the functional role of CAFs and their EVs in lung metastatic progression of SACC, SACC-LM cells were transplanted with or without CAF-A1/A2 into subcutaneous space of BALB/c nude mice that were raised for 3, 5 or 12 weeks **(**Fig. 6a**)**. After 3 weeks, no metastasis was found in the lungs of mice with any xenografts, but the expression FN, MMP9 and LOX, and POSTN could be recognized in the lungs (Additional file 2: Figure. S9). Especially, POSTN expression were significantly higher in the SACC+ CAF-A1/A2 groups than in the SACC-LM group and controls, indicating the pre-metastatic niche formation in the lungs of SACC+ CAF-A1/A2 groups (Fig. 6b **and** Additional file 2: Figure. S9). It suggests that CAFs promote the pre-metastatic niche formation in the lungs via their EVs since POSTN is a potential biomarker characterizing the CAF EV-induced pre-metastatic niche. After 5 weeks, SACC-LM+ CAF-A1/A2 xenografts were larger than SACC-LM xenografts and contained more blood vessels (Additional file 2: Figure. S10). Micrometastasis could be detected in the lungs of mice with SACC-LM+ CAF-A1/A2 xenografts (Additional file 2: Figure. S11). Statistical analyses demonstrated that both metastatic colony numbers and area were significantly higher in the SACC-LM + CAF-A1/A2 groups than in the SACC-LM group **(**Fig. 6b**)**. By 12 weeks, survival analysis demonstrated that mice with SACC-LM + CAF-A1/A2 transplantation had lower survival times than those with SACC-LM transplantation solely **(**Fig. 6b**)**. Taken together, transplantation of CAFs promoted the pre-metastatic niche formation at the early stage, leading to lung metastasis and poor prognosis of SACC.

Then we analyzed integrin β1-positive plasml EVs in the mice burden xenografts at the early and late stages. Plasma EVs were isolated and characterized (Additional file 2: Figure. S12B). In the pre-metastatic animal models, plasma EV integrin β1 could be detected in all the mice with xenografts, but not in the controls. Notably, integrin β1 expression was higher in the SACC-LM+ CAF-A1/A2 groups than in the SACC-LM group and controls **(**Fig. 6c**)**. We further analyzed the plasma EV integrin β1 using the metastatic animal models. Integrin β1-positive EVs in plasma sustained high level in the SACC-LM+ CAF-A1/A2 groups, compared to the SACC-LM group and controls **(**Fig. 6d**)**. As we have demonstrated that integrin β1 showed high expression in CAF EVs and low expression in SACC-LM EVs (Additional file 2: Figure. S7A), these integrin β1-positive plasma EVs were principally derived from CAF-A1/A2 cells. These results confirmed our hypothesis that CAF-derived integrin β1-positive EVs traveled to the lungs and induced the pre-metastatic niche formation, leading to the lung metastasis.

## Discussion

CAFs are known to be regulators of cancer invasion, metastasis and drug resistance via activating the crosstalk between cells, such as cancer cells, endothelial cells and immunocytes, in primary tumors [38–42]. In this study, we demonstrated, for the first time, that CAFs promoted metastasis by constructing a pre-metastatic niche in the lungs via their EVs. CAF EVs principally detected LFs via integrin α2β1 and consequently activated TGF-β signaling pathway in LFs. These activated fibroblasts remodeled the lung microenvironment to facilitate tumor cell colonization **(**Fig. 6e**)**.

Since the existence of the pre-metastatic niche was first demonstrated in 2005, various tumor-shed soluble factors and EVs have been detected to promote pre-metastatic niche formation [8, 18, 43]. Multiple events are involved in the process of pre-metastatic niche formation, including ECM remodeling and BMDC recruitment. However, to date, limited information has been available regarding the role of stromal cells in the process of pre-metastatic niche evolution. We hypothesize that the pre-metastatic niche may be generated as a result of the combined systemic effects of primary tumor microenvironment. CAF EVs may participate in the crosstalk between the primary tumor microenvironment and pre-metastatic organs. Here we showed that CAF EVs promoted pre-metastatic niche formation in a different way to tumor EVs. CAF EVs was found to be more capable of ECM remodeling than SACC EVs. CAF EVs targeted LFs and transformed them via TGF-β signaling pathway, leading to enhanced ECM remodel. Notably, the components of the ECM modified by CAF EVs and SACC EVs in the pre-metastatic niche were different. Among them, POSTN might be a specific marker of a CAF EV-constructed niche. CAF EVs induced overexpression of POSTN in LFs in vitro and in vivo at an early stage, which differed from SACC EVs. POSTN is a non-structural ECM protein that binds to collagen I and FN, regulating the interaction between cells and ECM [44, 45]. In recent years, POSTN was found to be involved in metastatic colonization by activating the AKT and STAT3 signaling pathways, promoting recruitment of myeloid-derived suppressor cells and creating an immune inhibitory environment in secondary organs [46]. Our result that CAF EVs induced LFs to produce POSTN may explain why the mice with transplantation of SACC cells and CAFs had a high risk of lung metastasis in our study.

Protein analysis demonstrated that both proteins stimulating and inhibiting TGF-β signaling pathway were present in CAF EVs. As we have demonstrated that CAF EVs could activate TGF-β signaling pathway in LFs, we considered that the multiple components in EVs may function in a complex network in target cells. Thus, it should be a good strategy to inhibit CAF EV-induced pre-metastatic niche formation by suppressing CAF EV uptake by target cells. EV integrins are known to determine organotropism. Specifically, EV integrins α6β4 and α6β1 have been associated with lung metastasis, whereas EV integrin αvβ5 has been linked to liver metastasis [29]. Our study demonstrated that lung tropic CAF EV expressed integrin α2β1 and their target cells were LFs. Downregulation of integrin β1 impacted CAF EV uptake by LFs and the CAF EV-induced pre-metastatic niche formation in the lungs. TC I-15, an inhibitor of integrin α2β1, reduced CAF EV uptake by LFs and consequently p-Smad3 and POSTN expression. In vivo, TC I-15 hindered ECM remodeling in the lungs and subsequent metastasis. Furthermore, we demonstrated that plasma EV integrin β1 increased before SACC lung metastasis in mice. Collectively, our data suggested that plasma EV integrin β1 represents a candidate biomarker for predicting the prognosis of SACC metastasis and directing anti-metastatic therapies.

In summary, we demonstrated that stromal cells in primary tumors, as well as tumor cells, could communicate with distant organs via their EVs and promote pre-metastatic niche formation in distant organs. CAF EVs showed superiority in ECM remodeling comparison with SACC EVs, and POSTN might be a biomarker for detecting a CAF EV-constructed pre-metastatic niche. Integrin α2β1 mediated CAF EV uptake by LFs and consequently activated the TGF-β signaling pathway in LFs. This study provides new insight into pre-metastatic niche formation. It also prompts the development of an integrated strategy targeting EVs from both tumor and stromal cells to prevent metastasis at an early stage.

## Supplementary information

**Additional file 1:** Supplementary methods, including TEM; NTA analysis; Assessment of EV distribution in vivo; EV proteomics; Western blot analysis; RNA sequencing; siRNA transfection; Wound healing assay; Collagen contraction assay; SACC-LM-GFP cells; Immunohistochemical staining; MVD evaluation; CCK8 assay; TUNEL assay.

**Additional file 2: **Supplementary figures, including **Figure S1**. Characterization of EVs; **Figure S2.** Representative images of the distribution of EVs from SACC-LM, CAF-A1 and CAF-A2 cells in the lungs, liver and brain of mice; **Figure S3**. ECM remodeling in the lungs at days 7 and 14 after EV treatment; **Figure S4.** EVs were taken up by specific cell types in the lungs; **Figure S5.** Phenotype changes were examined in lung fibroblasts treated with indicated EVs; **Figure S6.** LFs were not activated by NF EVs; **Figure S7.** Integrin β1 expression functionally contributed to CAF EV uptake by LFs in vitro and mediated pre-metastatic niche formation in the lungs; **Figure S8.** Cytotoxicity assays of TC I-15; **Figure S9.** Lung examination to confirm the pre-metastatic niche formation in the nude mice with indicated xenografts for 3 weeks; **Figure S10.** Xenograft examination after subcutaneous transplantation for 5 weeks; **Figure S11.** Lung examination after subcutaneous transplantation for 5 weeks; **Figure S12.** Mouse plasma EV characterization.

**Additional file 3: **Supplementary tables, including **Table S1**. GO enrichment analysis of Cellular Component based on RNA-Seq data; **Table S2.** GO enrichment analysis of Molecular Function based on RNA-Seq data; **Table S3.** GO enrichment analysis of Cellular Component based on ITRAQ data; **Table S4.** GO enrichment analysis of Molecular Function based on ITRAQ data.

## Data Availability

All the data generated or analyzed during this study are included in this published article and its supplementary files. The datasets and materials in the current study available from the corresponding author on reasonable request.

## Abbreviations

BMDCs: Bone marrow-derived cells
CAFs: Carcinoma-associated fibroblasts
CK: Cytokeratin
ECM: Extracellular matrix
EVs: Extracellular vesicles
FAP: Fibroblast activation protein
FN: Fibronectin
FSP1: Fibroblast-specific protein 1
GO: Gene ontology
LFs: Lung fibroblasts
LOX: Lysyl oxidase
LOXL2: Lysyl oxidase-like 2
MMPs: Matrix metalloproteinases
MVD: Microvessel density
NFs: Normal fibroblasts
POSTN: Periostin
SACC: Salivary gland adenoid cystic carcinoma
TEM: Transmission electron microscopy
TGF-β: Transforming growth factor-beta
TSP1: Thrombospondin-1
VEGFR1: Vascular endothelial growth factor receptor-1
α-SMA: Alpha-smooth muscle actin
