# Supplementary material for: Extracellular vesicles of carcinoma-associated fibroblasts creates a pre-metastatic niche in the lung through activating fibroblasts
Source: Mol Cancer. 2019 Dec 3;18:175. doi: 10.1186/s12943-019-1101-4 (PMC6892147; doi:10.1186/s12943-019-1101-4)
Supplement: Supplementary file 1 — Additional file 1: Supplementary methods, including TEM; NTA analysis; Assessment of EV distribution in vivo; EV proteomics; Western blot analysis; RNA sequencing; siRNA transfection; Wound healing assay; Collagen contraction assay; SACC-LM-GFP cells; Immunohistochemical staining; MVD evaluation; CCK8 assay; TUNEL assay. [file 12943_2019_1101_MOESM1_ESM.docx]

**­­Extracellular Vesicles of Carcinoma-associated Fibroblasts Creates a Pre-metastatic Niche in the Lung through Activating Fibroblasts**

**Supplementary methods**

**Transmission electron microscopy (TEM)**

TEM imaging was performed to characterize the morphology and size of EVs. Briefly, purified EVs were placed onto 200 mesh copper grids and incubated at room temperature for 15 min. Then the grids were dried and EVs were counterstained with phosphato-tungstic acid for 5 min. Images were recorded using an electron microscope (GEM-2000EX, Japan Electronics, Japan).

**Nanoparticle tracing system (NTA) analysis**

EVs isolated from the culture media and plasma were resuspended in PBS. The EV concentration and size distribution were analyzed using ZetaView (PERTICLE METRIX, German).

**Assessment of EV distribution *in vivo***

To assess EV distribution in the lungs, liver and brain, PKH67-labeled EVs were injected into the tail vein or the retro-orbital venous sinus of C57BL/6J mice. After 24 h, mice were euthanized and the lungs, liver and brain were fixed and embedded into OCT. Sectioned with 8 μm thickness were prepared and stained with diamidino-2-phenylindole (DAPI). Sections were mounted with fluorescence mounting media and visualized using an inverted microscope. To detect the EV target cells in the lungs, sections were blocked with 10% goat serum and stained with collagen I (1:500, Abcam), FSP1 (1:200, Proteintech), EpCAM (1:200, Proteintech), and CD34 (1:150, Abcam). Nuclei were counterstained with DAPI.

**EV proteomics**

EV protein was extracted by RIPA (R0010, Solarbio, China) with protease inhibitor and submitted for analysis using ITRAQ (BGI Company, China). Two comparison groups were set up as follows: CAF-A1 vs. SACC-LM, CAF-A2 vs. SACC-LM. EV proteins were digested with trypsin at 37°C for 8 h. Then the peptides were dried and dissolved with 0.5 M triethylamine-carbonate buffer. An ITRAQ Reagent 8-plex Kit (SCIEX, Massachusetts, US) was used for the ITRAQ labeling of peptide samples. The labeled peptides were fractionated using the reverse phase. All of the ITRAQ data were obtained using the TripleTOF 5600 System (SCIEX).

**Western blot analysis**

Total cell or EV proteins were extracted by RIPA and quantified with a Bradford Protein Assay Kit (Beyotime, Beijing, China). Samples (20 μg) were mixed with SDS-PAGE Sample Loading Buffer (Beyotime) and separated on 10% or 8% SDS-PAGE gels, then transferred to nitrocellulose membrane (Merck Millipore). After blocking with 3% BSA, membranes were incubated with the appropriate primary antibodies, including CD63 (1:500, Abcam), CD9 (1:500, Abcam), CD81 (1:200, BOSTER), HSP70 (1:500, Abcam), CALNEXIN (1:500, Proteintech), p-Smad 2 (1:2000, Cell Signaling), p-Smad 3 (1:1000, Abcam), LOXL2 (1:500, Abcam), MMP2 (1:500, Abcam), TSP1 (1:500, Proteintech), POSTN (1:500, Abcam), Integrin β1 (1:1000, Abcam), Integrin α2 (1:1000, Abcam), Integrin α4 (1:1000, Abcam) and Integrin α6 (1:2000, Abcam). Antibodies angainst β-actin (1:1000, Proteintech, Illinois, US) or GAPDH (1:2000, Proteintech) were used as controls. After incubation with a HRP-conjugated anti-rabbit or anti-mouse secondary antibody (1:2000, Bioss, Beijing, China), the intensity of the immunoreactive proteins was visualized with the ECL detection system (Beyotime).

**RNA sequencing**

Mouse primary LFs in 6-well plate were treated with EVs from CAF-A1 and CAF-A2 for 3 days. LFs without EV treatment were used as a control. Total RNA was extracted from LFs using TRIzol™ Reagent (Thermo). RNA sequencing was performed by Novogene Bioinformatics Technology Co. Ltd (Shanghai, China). In brief, RNA quality control was examined by 1% agarose gel electrophoresis, spectrophotometry (NanodropTM 1000, Thermo) and Agilent Technologies 2100 Bioanalyzer. Then, mRNA was converted into a library of cDNA fragments and sequencing using an Illumina HiSeq 2000 sequencer. All procedures were performed following the manufacturer’s instructions.

**siRNA transfection**

To downregulate TSP1 expression, two pairs of TSP1-specific siRNA (siTSP1-1: CCA CAG GCC AAA GAC GGG UUU CAU U/A AUG AAA CCC GUC UUU GGC CUG UGG; siTSP1-2: UGG CAU CCC UGA GGC AGA UGA AGA A/U UCU UCA UCU GCC UCA GGG AUG CCA) and a non-targeting siRNA (siNC: UUC UCC GAA CGU GUC ACG UTT ACG UGA CAC GUU CGG AGA ATT) were used. Integrin β1-specific siRNA (si-integrin β1: GCA CCA GCC CAU UUA GCU ATT/ UAG CUA AAU GGG CUG GUG CTT) and a non-targeting siRNA (siNC: UUC UCC GAA CGU GUC ACG UTT/ ACG UGA CAC GUU CGG AGA ATT) were used to knockdown integrin β1 expression. These products were purchased from Genepharma (Shanghai, China). Cells (1.0 × 10^6^ per well) were seeded in 6-well plates and transfected for 10 h with 20 nM siRNA using Lipofectamine™ 2000 (Thermo, USA) according to the manufacturer’s protocol.

**Wound healing assay**

Cells in FBS-free medium were seeded into two square wells (4×10^5^ cells/well) with 1 mm spacing in a polydimethylsiloxane (PDMS) membrane that was bonded in a 6-well plate in triplicate. Each well in the PDMS membrane is a square with sides 10 mm in length and 5 mm in height. Cells reached confluency in the two wells after 24 h. Then the PDMS membrane was peeled off and a wound (1 mm in width) was formed. Cells were cultured in FBS-free medium and treated with EVs (250 μg) from SACC-LM or CAF-A1/A2 for 8 h. Serum-free DMEM/F12 was used as a control. The wound area was recorded using an inverted microscope and calculated using Image-Pro Plus 6.0 (Rockville, MD, USA).

**Collagen contraction assay**

A total of 2 × 10^5^ LFs were suspended in 100 μL medium. Then the cell suspension was mixed with 100 μL of collagen containing 68.75 μL medium, 0.72 μL NaOH (1 N), and 31.25 μL Rat Tail Collagen I (Corning), and added to 1 well of 24-well plates in triplicate and allowed to solidify for 45 min at 37 °C. After incubation with media containing EVs (25μg) for 24 h, the gels were photographed. ImageJ software was used to measure gel area and evaluate contraction.

**SACC-LM-GFP cells**

GFP-labeled SACC-LM cells were obtained by lentivirus infection. Expression plasmid (pEX-GFP) and packaging plasmids (psPAX2 and pMD2.G) were purchased from GenePharma Co., Ltd (China). 293T cells were co-transfected with the pEX-GFP, psPAX2 and pMD2.G vectors using Lipofectamine™ 2000 Transfection Reagent (ThermoFisher Scientific). Then, lentiviruses were harvested from the 293T cells in viral harvesting medium by centrifugation 2500 *g* and used to infect SACC-LM cells. Puromycin (2 μg/mL, Sigma) was applied to select GFP-positive cells.

**Immunohistochemical staining**

Immunohistochemical staining of paraffin-embedded human SACC tissues was performed on 4-μm-thickness sections using SPlink Detection Kits (SP-9000, ZSGB-BIO, China). The sections were deparaffinized and rehydrated. Endogenous peroxidase activity was blocked with 3% hydrogen peroxide in methanol. Nonspecific binding sites were blocked with 10% goat serum. Sections of human SACC tissues were incubated with FAP (1:200, Abcam) overnight at 4°C. Immunoreactions were detected using 3, 3ʹ-diaminobenzidine as the final chromogen. Nuclei were counterstained with hematoxylin. Negative control experiments were carried out by replacing the primary antibody with PBS. The integrated optical density and area of target distribution were measured with Image-Pro® Plus version 6.0. We calculated the mean density of each field, and took the average of the mean density of at least 10 fields as the mean density of each case.

To confirm large metastatic colonies in the lungs of nude mice with SACC-LM xenograft, the lungs embedded in OCT were sectioned into 8 μm thick slices. Immunohistochemical staining was performed using SPlink Detection Kits (SP-9001, ZSGB-BIO, China). Briefly, the sections were blocked with 10% goat serum and incubated with anti-human pan CK (Merck Millipore) overnight at 4°C. Immunoreactions were detected using 3, 3ʹ-diaminobenzidine as the final chromogen. Nuclei were counterstained with hematoxylin. Negative control experiments were carried out by replacing the primary antibody with PBS.

**MVD evaluation**

SACC-LM xenografts with or without CAF-A1/A2 were sectioned at a thickness of 8μm. Immunofluorescent staining with anti-CD34 antibody (1:200, Abcam) was performed. Immunoreactions were detected with FITC-labeled goat anti-rabbit secondary antibody (1:200, Abbkine). Nuclei were counterstained with DAPI. MVD was evaluated by counting CD34-labeled vessels in the endothelium. Hot spots (intense neovascularization) were assessed in tumor areas showing the highest density of CD34 staining. For vessel counting, a high-power field (× 400) in each of the 10 hot spots was used. A CD34-positive vessel or endothelial cell cluster that was clearly separate from adjacent microvessels, tumor cells and other connective tissue elements was considered to be a single, countable microvessel. A vessel lumen was not required for identification of a microvessel. Macrovessels, characterized by thick muscular walls or with lumina greater than eight red blood cells in diameter (approximately 50 μm), were excluded from the count.

**CCK8 assay**

LFs were seeded in a 96-well plate (5×10^3^cells/well) and cultured for 24, 48, and 72 h. CCK8 reagent (Biosharp, China) mixed with DMEM F/12 medium (10 μL+ 90 μL) was added in each well and incubated for 1 h before the end of the culture time. Then, the absorbance at 450 nm was measured using a microplate reader (Bio-Rad, Hercules, CA, USA). Each well was repeated at least 3 times, and the mean absorbance was used to assess cell proliferation.

**TUNEL assay**

Cell apoptosis examination was performed by TUNEL assay. TUNEL Apoptosis Detection Kit (FITC) was purchased from YEASEN Co., Ltd (Shanghai, China). LFs were seed in a 24-well plate and cultured for 72 h. Triton X-100 (0.2%) was applied to the LFs for 5 min. Then the cells were incubated with 1× Equilibration Buffer for 30 min. Mixture of FITC-12-dUTP Labeling Mix and Recombinant TdT Enzyme was prepared according to the manufacturer’s protocol. The mixture (100 μL) was added in each well and incubated at 37℃ for 60 min. After washing with PBS containing 5 mg/mL BSA and 0.1% Triton-X100 for 5 min, the NFs were counterstained with DAPI.
