# Supplementary material for: Extracellular vesicles of carcinoma-associated fibroblasts creates a pre-metastatic niche in the lung through activating fibroblasts
Source: Mol Cancer. 2019 Dec 3;18:175. doi: 10.1186/s12943-019-1101-4 (PMC6892147; doi:10.1186/s12943-019-1101-4)
Supplement: Supplementary file 2 — Additional file 2: Supplementary figures, including Figure S1. Characterization of EVs; Figure S2. Representative images of the distribution of EVs from SACC-LM, CAF-A1 and CAF-A2 cells in the lungs, liver and brain of mice; Figure S3. ECM remodeling in the lungs at days 7 and 14 after EV treatment; Figure S4. EVs were taken up by specific cell types in the lungs; Figure S5. Phenotype changes were examined in lung fibroblasts treated with indicated EVs; Figure S6. LFs were not activated by NF EVs; Figure S7. Integrin β1 expression functionally contributed to CAF EV uptake by LFs in vitro and mediated pre-metastatic niche formation in the lungs; Figure S8. Cytotoxicity assays of TC I-15; Figure S9. Lung examination to confirm the pre-metastatic niche formation in the nude mice with indicated xenografts for 3 weeks; Figure S10. Xenograft examination after subcutaneous transplantation for 5 weeks; Figure S11. Lung examination after subcutaneous transplantation for 5 weeks; Figure S12. Mouse plasma EV characterization. [file 12943_2019_1101_MOESM2_ESM.docx]

**Extracellular Vesicles of Carcinoma-associated Fibroblasts Creates a Pre-metastatic Niche in the Lung through Activating Fibroblasts**

**Additional file 2: figures**


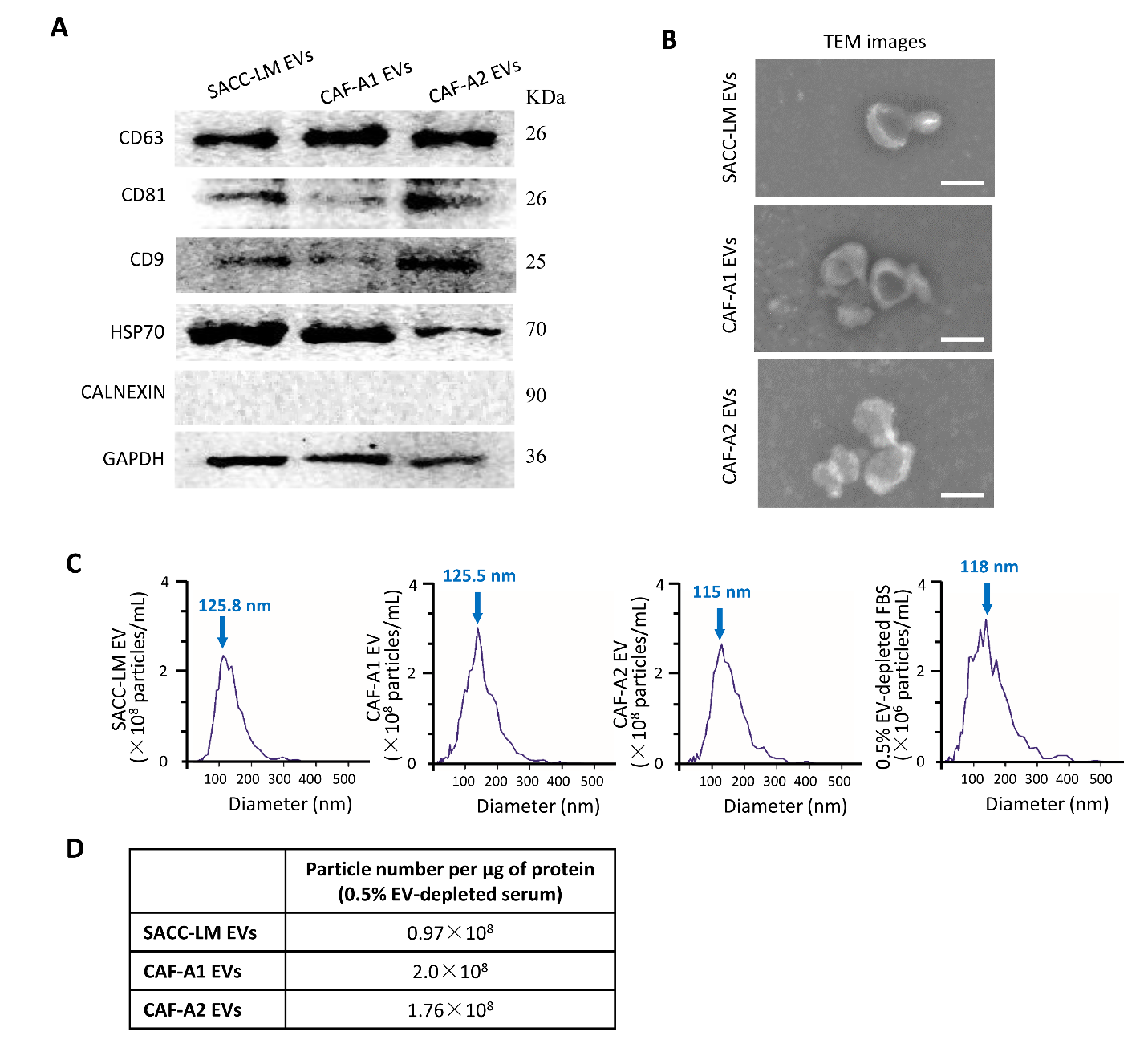


**Figure S1. Characterization of EVs.** (A) Western blot analysis confirmed the positive expression of CD63, CD81, CD9, and HSP70 and negative expression of CALNEXIN in EVs. (B) Representative TEM images of EVs from SACC-LM, CAF-A1 and CAF-A2 cells. Scale bar = 100 nm. (C) Particle concentration and size distribution in media containing 0.5% EV-depleted FBS after culturing indicated cells for 3 days. Medium containing 0.5% EV-depleted FBS before culturing cells was used as a control. (D) The particle number per μg of protein for each cell line was analyzed.


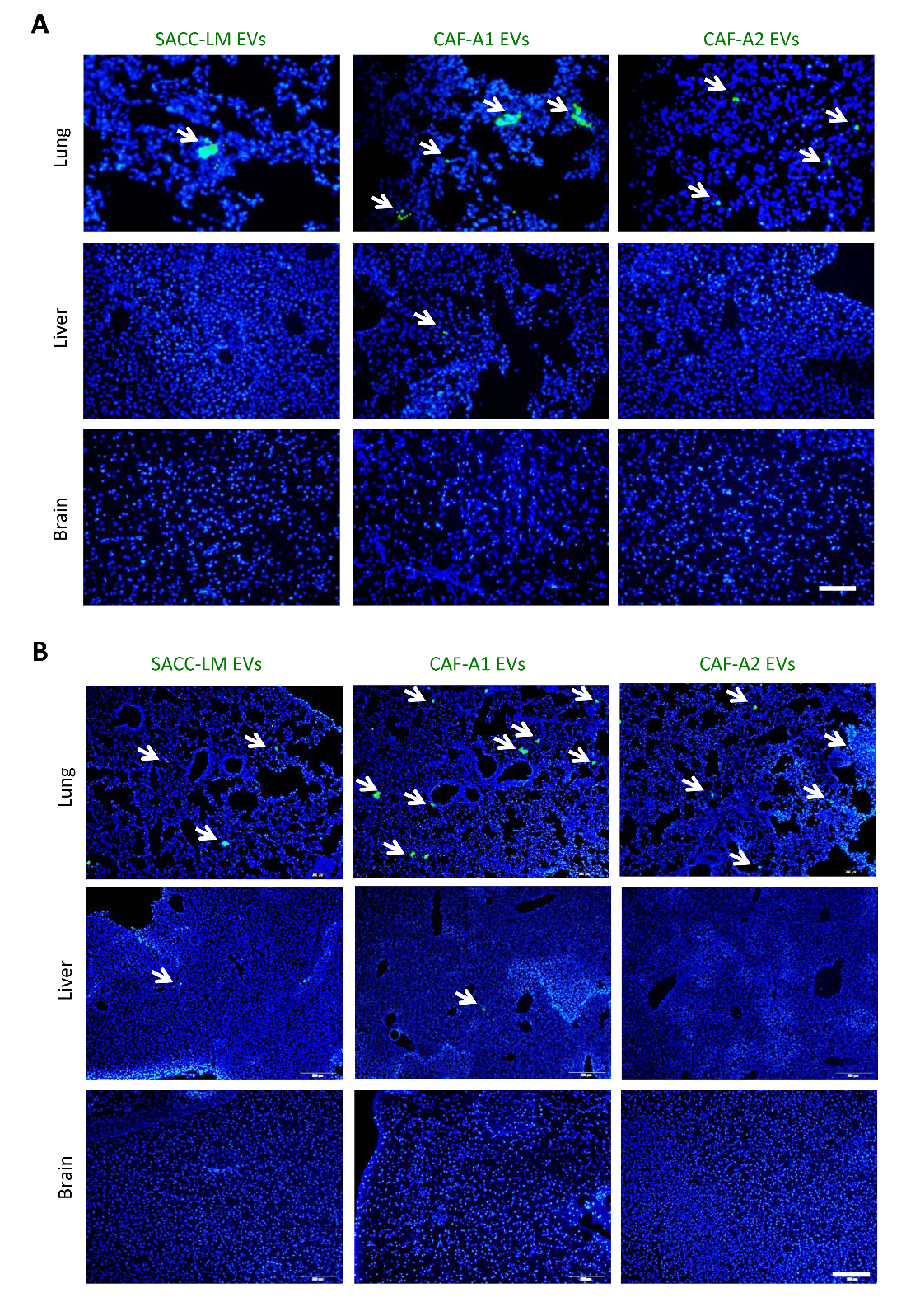


**Figure S2. Representative images of the distribution of EVs (green) from SACC-LM, CAF-A1 and CAF-A2 cells in the lungs, liver and brain of mice (n = 5 per group).** (A) EVs were injected into the tail vein. Scale bar = 50 μm. (B) EVs were injected into the retro-orbital sinus. Scale bar = 200 μm. EVs were found principally in the lungs (arrows), rarely in the liver, and not in the brain. In addition, there were more EVs in the lungs by tail vein injection than retro-orbital injection.


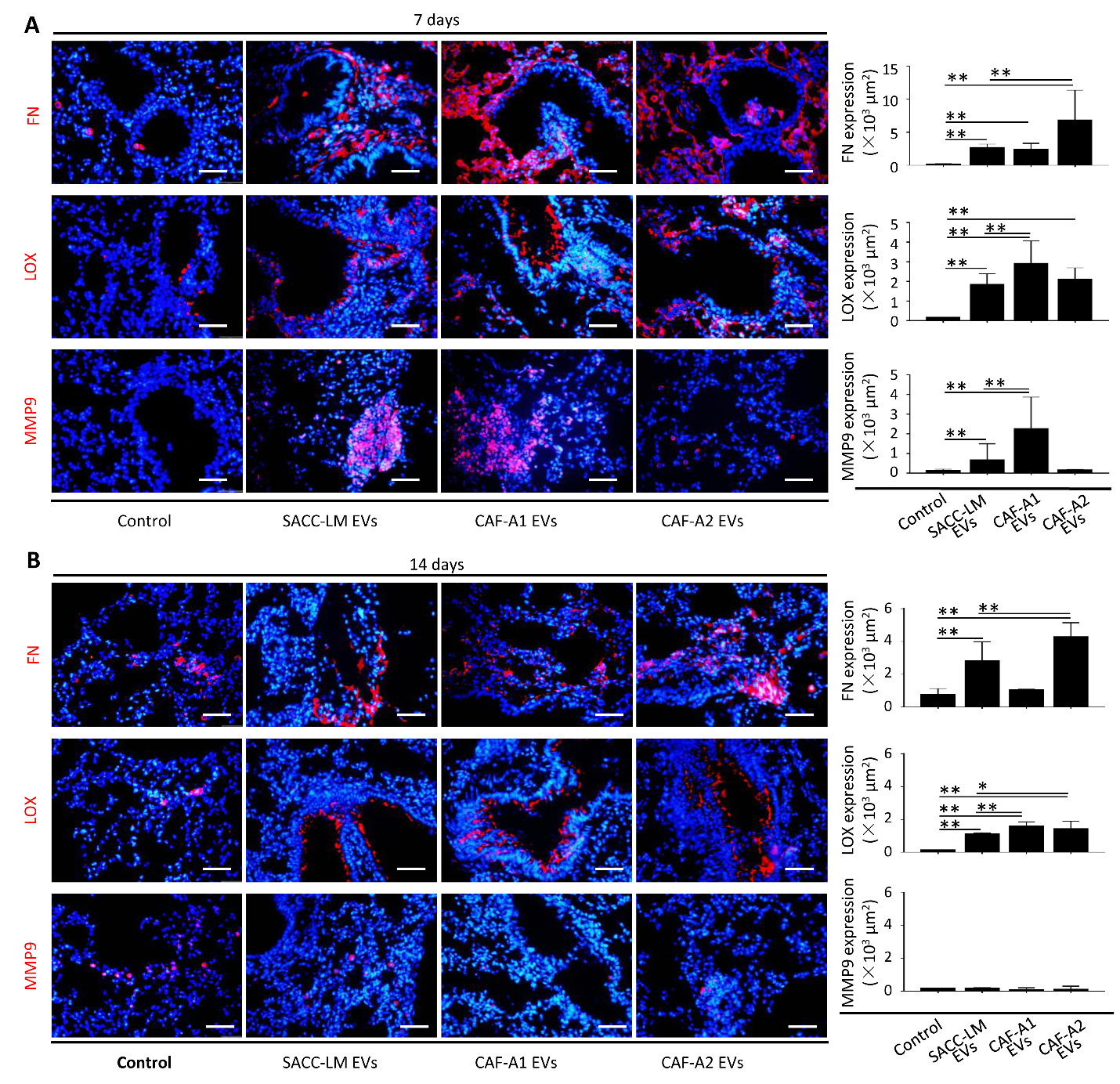


**Figure S3. ECM remodeling in the lungs at days 7 and 14 after EV treatment (n = 5 per group).** (A) Immunofluorescence staining and statistical analyses of FN, LOX, and MMP9 expression in the lungs at day 7 after the indicated EV treatment. (B) Immunofluorescence staining and statistical analyses of FN, LOX and MMP9 expression in the lungs at day 14 after the indicated EV treatment. Scale bar = 50 μm. * *P* <0.05, ** *P* <0.01.


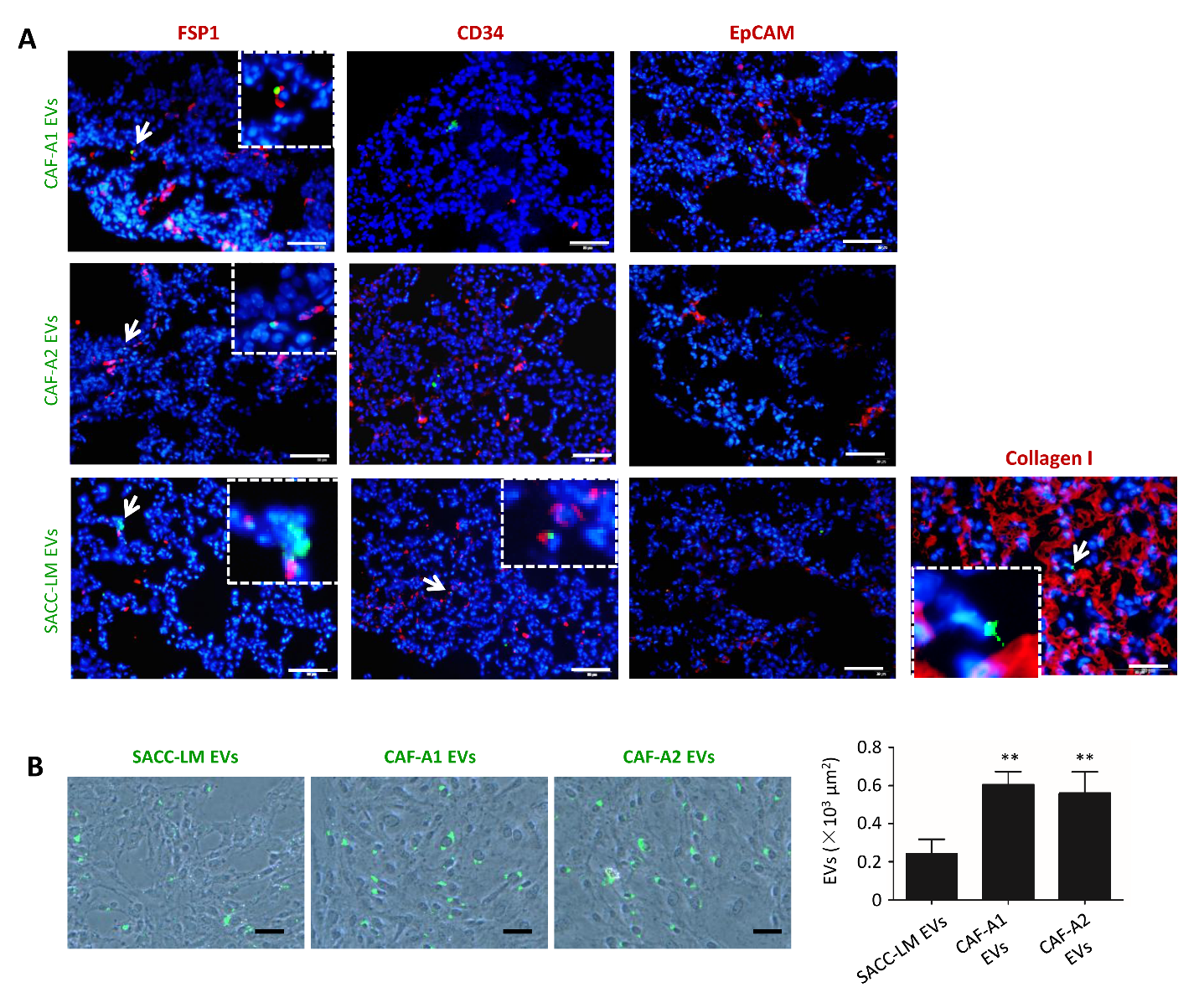


**Figure S4. EVs were taken up by specific cell types in the lungs.** (A) EVs were labeled with PKH67 (green) and the lung tissue sections were stained with FSP1, CD34, EpCAM and collagen I antibodies (red) to detect fibroblasts, endothelial cells or bronchial epithelial cells. CAF-A1/A2 EVs co-localized with FSP1-positive cells, but not CD34- or EpCAM-positive cells in the lungs. SACC-LM EVs did not co-localized with FSP1-, EpCAM-, or collagen I-positive cells. Co-localization of SACC-LM EVs with CD34-positive cells was found in the lungs. (n = 5). Scale bar = 50 μm. (B) Primary mouse LFs were incubated with PKH67 labeled EVs (green) from SACC-LM, CAF-A1 and CAF-A2 cells for 4 h. The amount of CAF-A1/A2 EVs uptake by LFs was more than that of SACC-LM EVs. Scale bar = 100 μm. ** *P* < 0.01, *** *P* < 0.001.


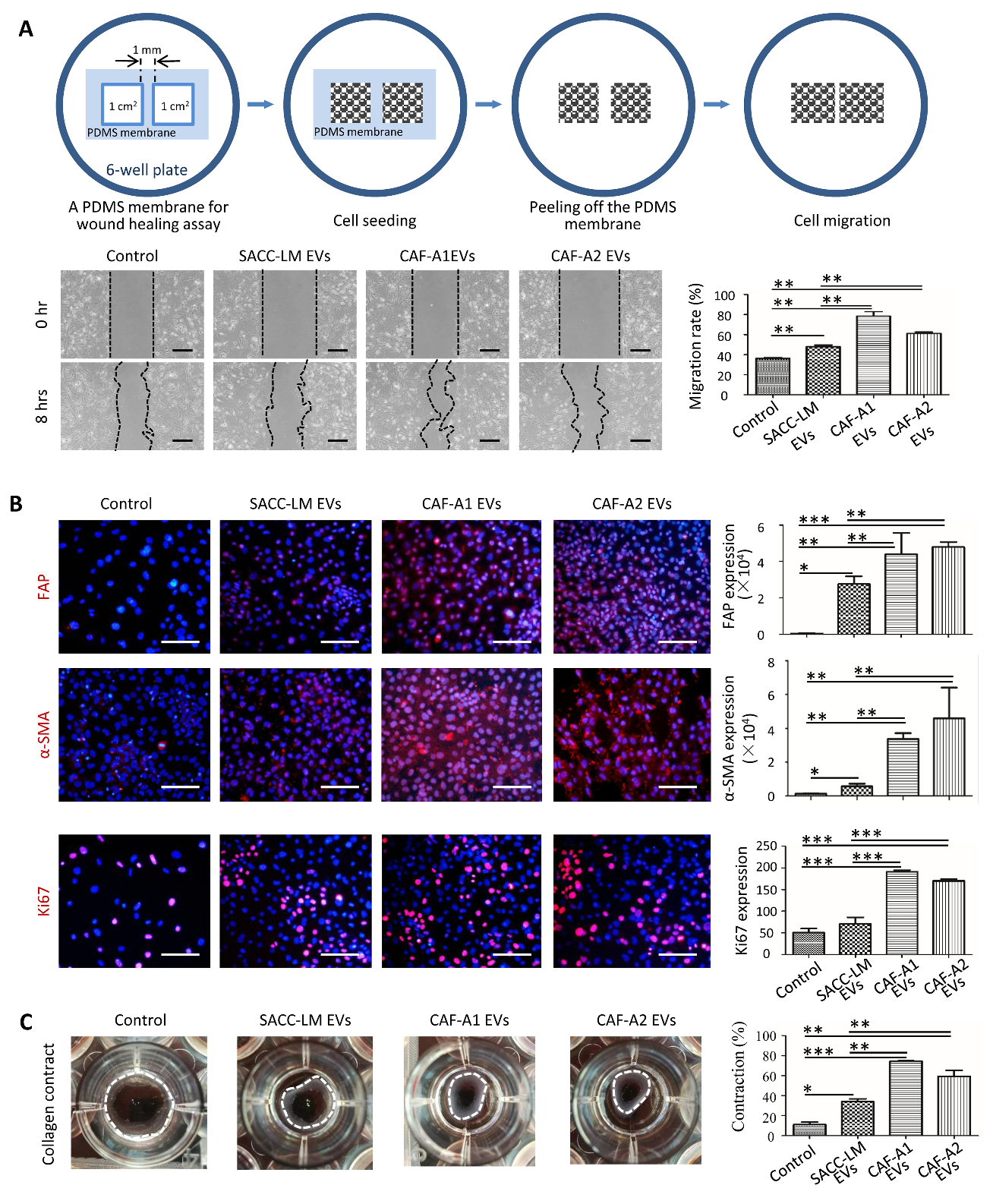


**Figure S5. Phenotype changes were examined in LFs treated with indicated EVs.** (A) Wound healing assay. Experimental steps using a PDMS membrane were illustrated. It was demonstrated that CAF-A1/A2 EVs increased the migration ability of LFs, compared with the control and SACC-LM EVs. Scale bar = 100 μm. (B) CAF-A1/A2 EVs also increased FAP, α-SMA, and Ki67 expression in the LFs, compared with the control and SACC-LM EVs. Scale bar = 50 μm. ** *P* < 0.01, *** *P* < 0.001. (C) Collagen gel contract was promoted by CAF-A1/A2 EVs, compared with SACC-LM EVs and the control. The EVs tested were 25 μg/well.


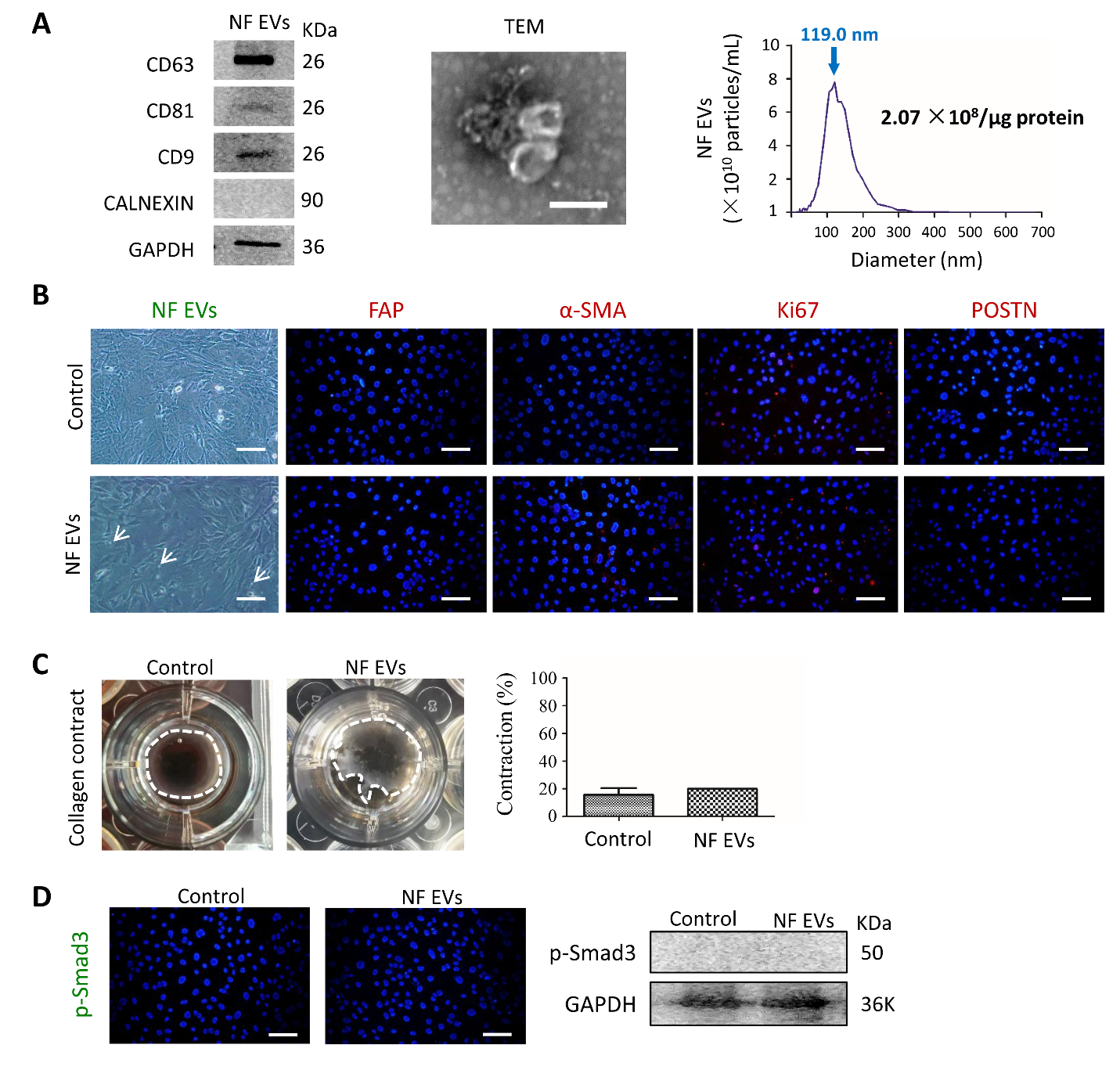


**Figure S6. LFs were not activated by NF EVs.** (A) The characteristics of NF EVs were confirmed by western blot analysis of CD63, CD81, CD9, and CALNEXIN expression, TEM image and NTA analysis. Scale bar = 100 nm. (B) NF EVs (green, arrows) were rarely internalized by LFs. Immunofluorescent staining demonstrated that NF EVs didn’t increase FAP, α-SMA, Ki67, and POSTN expression in LFs, compared with the control. Scale bar = 50 μm. (C) NF EVs (25 μg/well) didn’t promote collagen gel contract, compared with the control. (D) The expression of p-Smad3 in LFs didn’t changed by NF EV treatment. Scale bar = 50 μm.


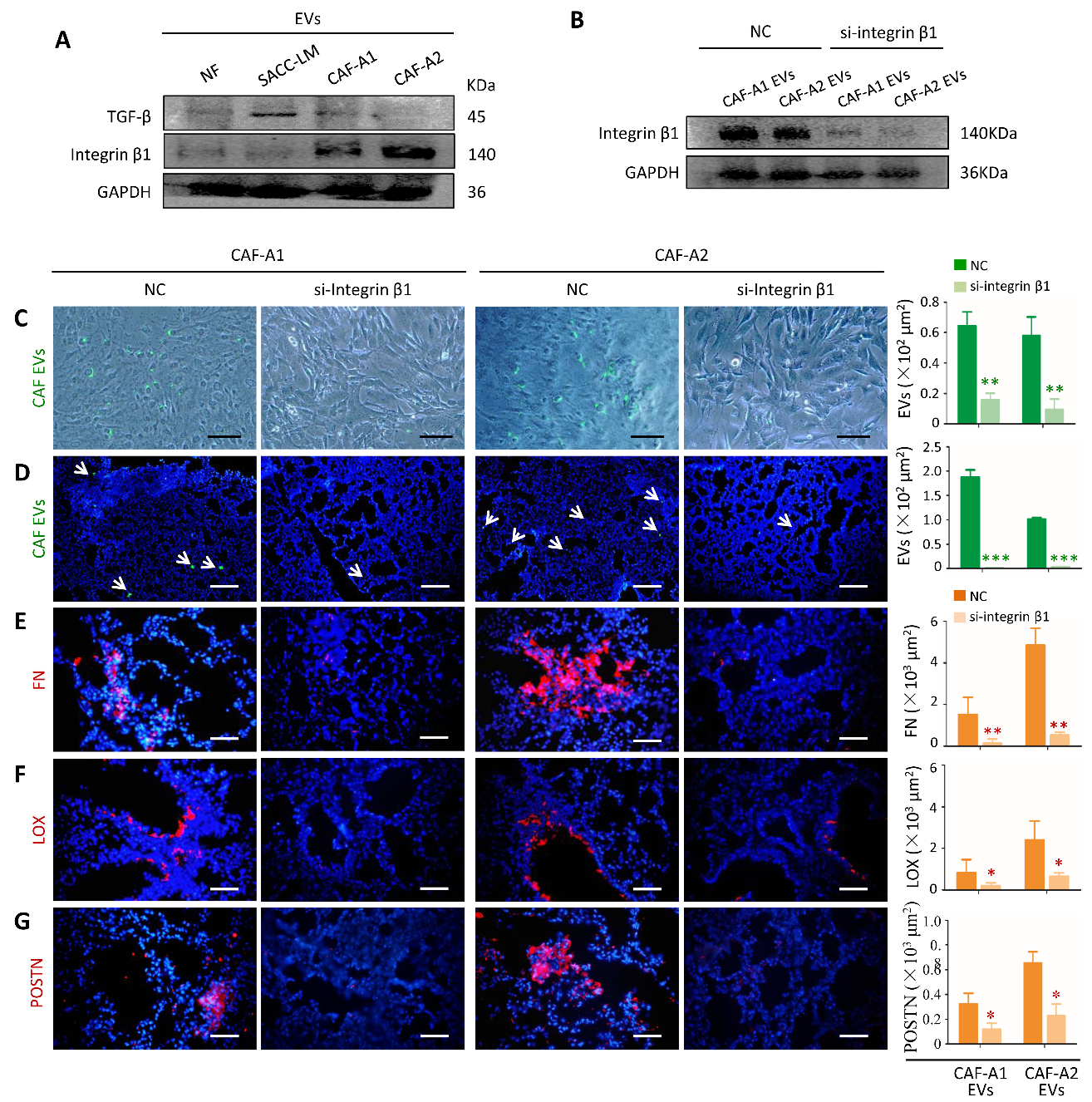


**Figure S7. Integrin β1 expression functionally contributed to CAF EV uptake by LFs in vitro and mediated pre-metastatic niche formation in the lungs.** (A) The expression of TGF-β and integrin β1 in NF, SACC-LM, CAF-A1, and CAF-A2 EVs by western blot analysis. (B) Downregulation of integrin β1 in CAF-A1/A2 EVs by integrin β1-specific siRNA transfection. (C) CAF-A1/A2 EVs-si-integrin β1 uptake by LFs in vitro. The amount of CAF-A1/A2 EVs-si-integrin β1 uptake by LFs decreased, compared to CAF-A1/A2 EVs. Scale bar = 100 μm. (D) Localization of CAF-A1/A2 EVs-si-integrin β1 in the lungs of mice. The amount of CAF-A1/A2 EVs-si-integrin β1 distributed in the lungs decreased, compared to CAF-A1/A2 EVs. Scale bar = 50 μm. (E-G) Expression of the pre-metastatic niche biomarkers in the lungs. Immunofluorescence staining and quantification demonstrated that the expression of FN (E), LOX (F), and POSTN (G) in the lungs significantly decreased in the CAF-A1/A2 EVs-si-integrin β1 groups, compared to the CAF-A1/A2 EV groups. Scale bar = 50 μm. * *P* <0.05, ** *P* <0.01.


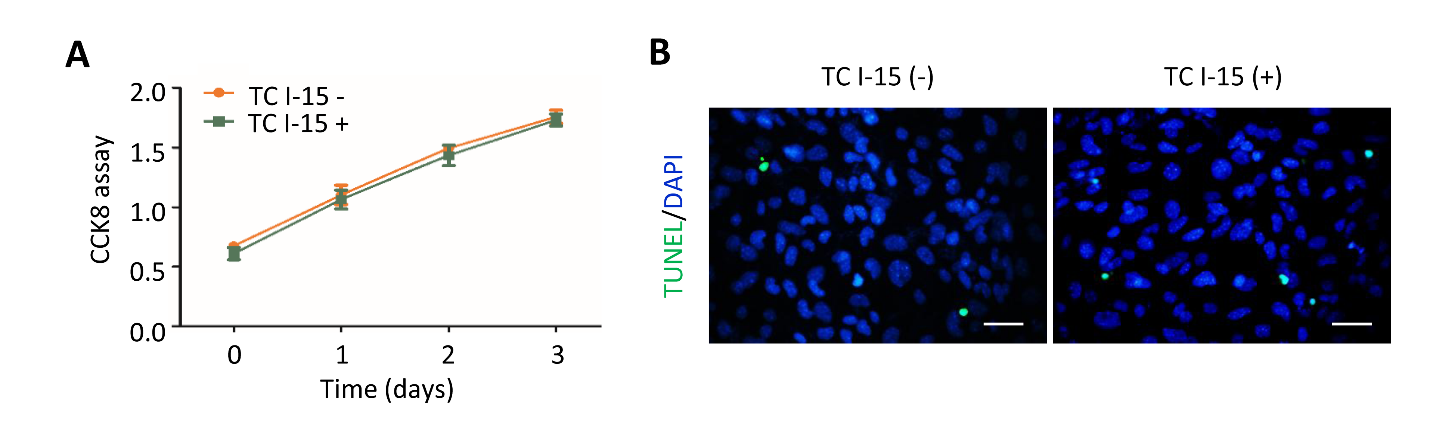


**Figure S8. Cytotoxicity assays of TC I-15. LFs were treated with TC I-15 (2μM) for 3 days.** (A) CCK8 assay demonstrated that LF proliferation didn’t changed by TC I-15 treatment. (B) TUNEL assay demonstrated that TC I-15 didn’t induce apoptosis in LFs. Scale bar = 100 μm.


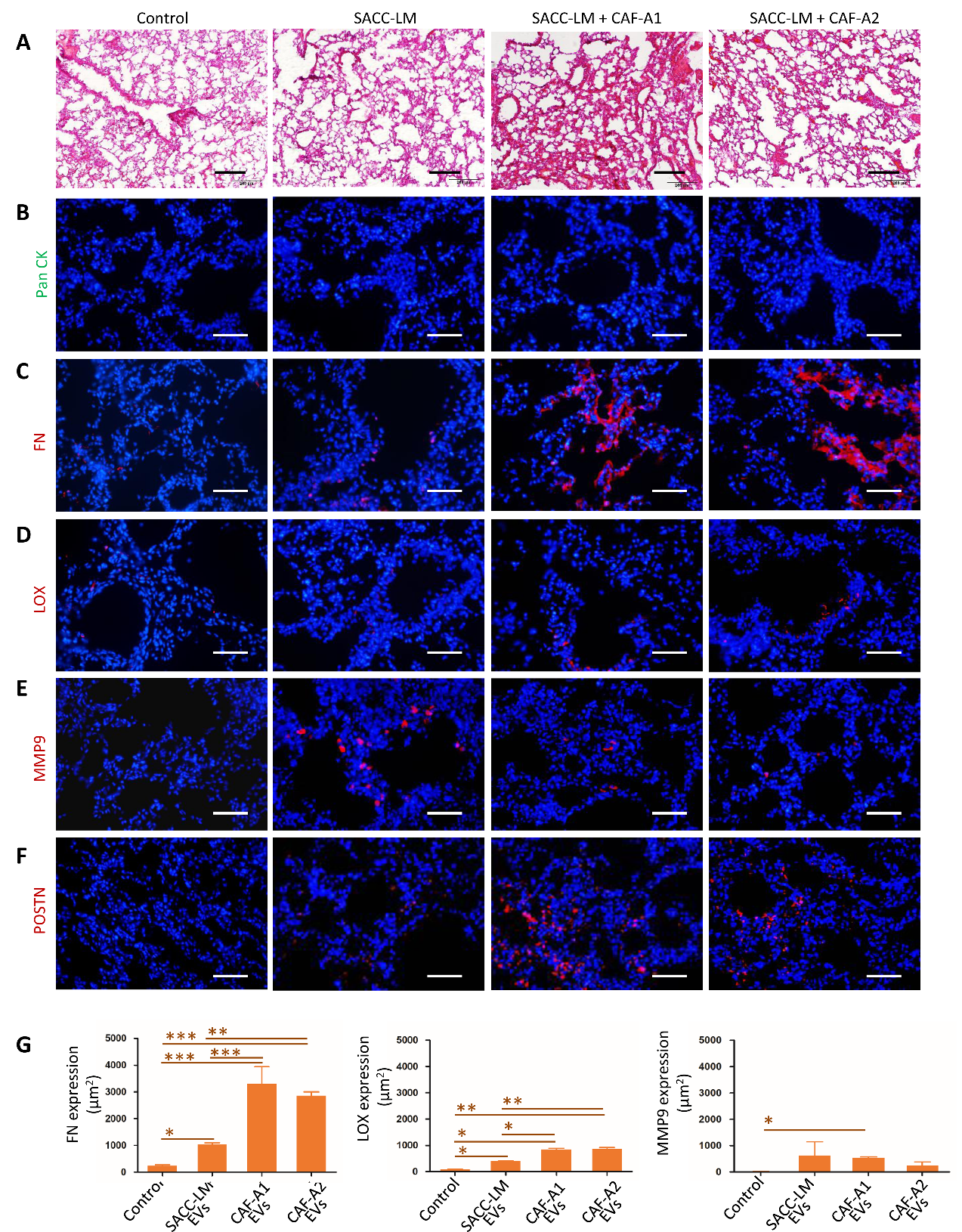


**Figure S9. Lung examination to confirm the pre-metastatic niche formation in the nude mice with indicated xenografts for 3 weeks (n = 5 per group).** (A) HE staining of the lung tissues. Scale bar = 200 μm. (B) Anti-human Pan CK staining found no metastasis in the lungs. (C-F) Expression of FN (C), LOX (D), MMP9 (E), and POSTN (F) in the lungs by immunofluorescence staining. Scale bar = 50 μm.(G) Quantification of FN, LOX, and MMP9 expressions in the lungs. * *P* <0.05, ** *P* <0.01, *** *P* <0.001.


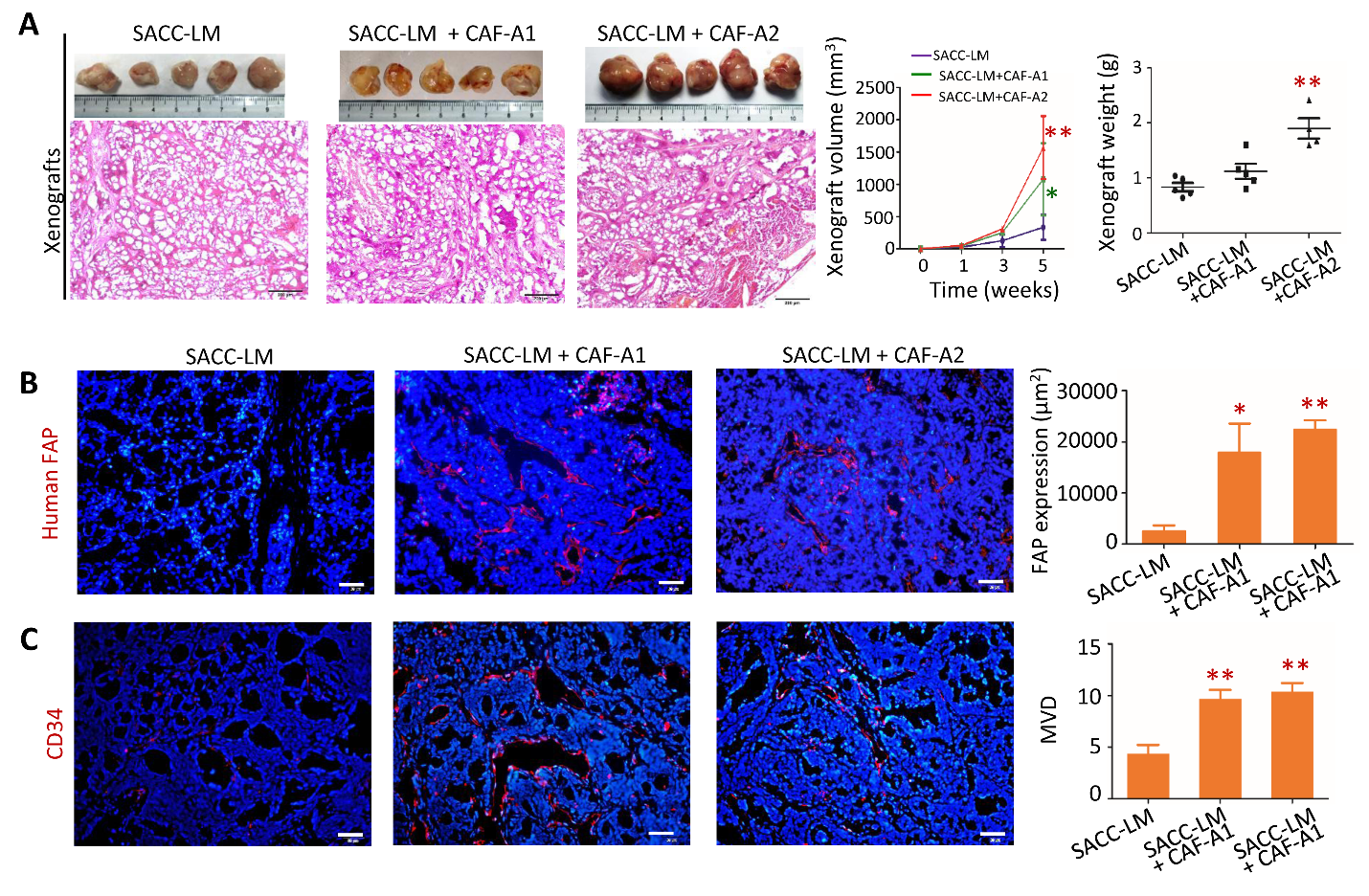


**Figure S10. Xenograft examination after subcutaneous transplantation for 5 weeks (n = 5 per group).** (A) HE staining of the indicated xenografts. All xenografts showed typical SACC histological architectures. Volume of SACC-LM+CAF-A1/A2 xenografts was larger than that of SACC-LM xenografts. Scale bar = 200 μm. (B) Immunofluorescent staining of the indicated xenografts using anti-human FAP. Cells with positive human FAP expression could be observed in the xenografts of SACC-LM + CAF-A1/A2, indicating the presence of CAF-A1/A2. Scale bar = 50 μm. (C) Immunofluorescent staining of lung tissues using anti-mouse CD34. The MVD was higher in the SACC-LM+CAF-A1/A2 xenografts than in the SACC-LM xenografts. High MVD in tumor tissues is commonly related with metastasis. Scale bar = 50 μm. * *P* <0.05, ** *P* <0.01.


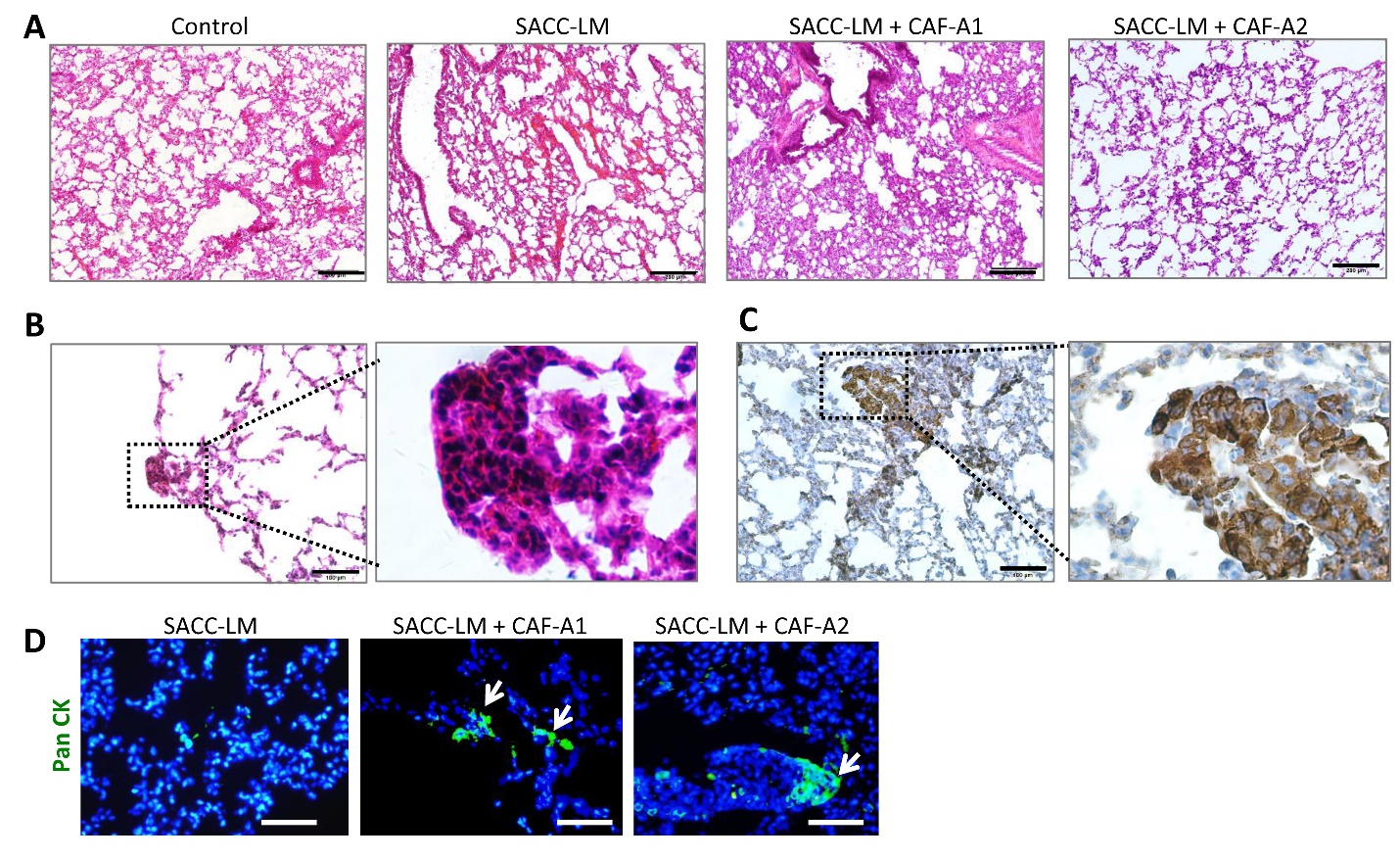


**Figure S11. Lung examination after subcutaneous transplantation for 5 weeks (n = 5 per group).** (A) HE staining revealed disorganized thickening of alveolar septae in the lung tissues of mice with xenografts, compared to the normal controls. Scale bar = 200 μm. (B) A typical micrometastasis could be observed at the lung surface in a nude mouse with SACC-LM + CAF-A2 xenograft. The right photo was the enlargement of the black rectangular area in the left photo. Scale bar = 100 μm. (C) Immunohistochemical stained lung tissues using anti-human pan CK. A micrometastasis with positive expression of anti-human pan CK (brown) could be recognized in the lung of a nude mouse with SACC-LM + CAF-A2 xenograft. The right photo was the enlargement of the black rectangular area in the left photo. Scale bar = 100 μm. (D) Immunofluorescent staining of the lung tissues with anti-human pan CK. Micrometastatic colonies (arrows) were detected in the SACC-LM + CAF-A1/A2 groups. No obvious micrometastasis was found in the SACC-LM group. Scale bar = 50 μm.


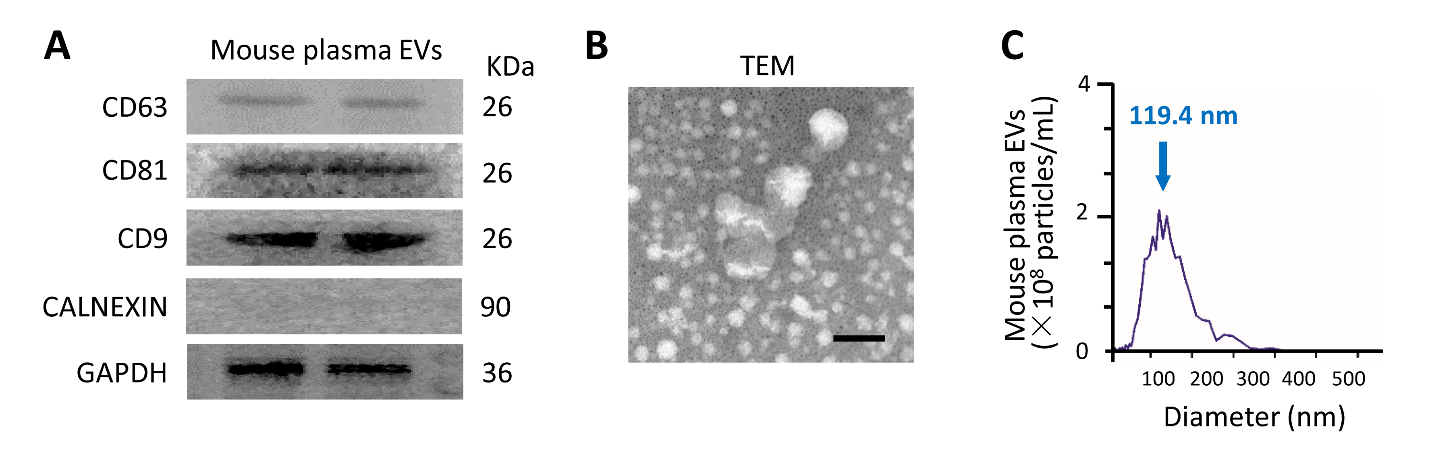


**Figure S12. Mouse plasma EV characterization.** (A) Western blot analysis confirmed the positive expression of CD63, CD81, and CD9 and negative expression of CALNEXIN. (B) TEM image. Scale bar = 100 nm. (C) NTA analysis.
