# Supplementary material for: Extracellular vesicles of carcinoma-associated fibroblasts creates a pre-metastatic niche in the lung through activating fibroblasts
Source: Mol Cancer. 2019 Dec 3;18:175. doi: 10.1186/s12943-019-1101-4 (PMC6892147; doi:10.1186/s12943-019-1101-4)
Supplement: Supplementary file 3 — Additional file 3: Supplementary tables, including Table S1. GO enrichment analysis of Cellular Component based on RNA-Seq data; Table S2. GO enrichment analysis of Molecular Function based on RNA-Seq data; Table S3. GO enrichment analysis of Cellular Component based on ITRAQ data; Table S4. GO enrichment analysis of Molecular Function based on ITRAQ data. [file 12943_2019_1101_MOESM3_ESM.docx]

**Extracellular Vesicles of Carcinoma-associated Fibroblasts Creates a Pre-metastatic Niche in the Lung through Activating Fibroblasts**

**Additional file 3: Tables**

Table S1. GO enrichment analysis of Cellular Component based on RNA-Seq data

| ID | Description | GeneRatio | *P* value |
| --- | --- | --- | --- |
| GO: 0005578 | Proteinaceous extracellular matrix | 116/1873 | 1.51E-34 |
| GO: 0031012 | Extracellular matrix | 142/1873 | 1.46E-28 |
| GO: 0044420 | Extracellular matrix component | 60/1873 | 3.44E-25 |
| GO: 0005604 | Basement membrane | 46/1873 | 9.11E-19 |
| GO: 0005581 | Collagen trimer | 37/1873 | 2.78E-13 |
| GO: 0098644 | Complex of collagen trimers | 13/1873 | 1.92E-07 |
| GO: 0005583 | Fibrillar collagen trimer | 9/1873 | 2.13E-05 |
| GO: 0098643 | Banded collagen fibril | 9/1873 | 2.13E-05 |
| GO: 0005605 | Basal lamina | 10/1873 | 0.002759 |
| GO: 0043235 | Receptor complex | 53/1873 | 0.002759 |
| GO: 0031594 | Neuromuscular junction | 18/1873 | 0.002782 |
| GO: 0045177 | Apical part of cell | 59/1873 | 0.002782 |
| GO: 0008305 | Integrin complex | 12/1873 | 0.005442 |
| GO: 0042383 | Sarcolemma | 27/1873 | 0.00866 |
| GO: 0098636 | Protein complex involved in cell adhesion | 12/1873 | 0.009798 |
| GO: 0071953 | Elastic fiber | 4/1873 | 0.010205 |
| GO: 0009897 | External side of plasma membrane | 41/1873 | 0.014523 |
| GO: 0043034 | Costamere | 8/1873 | 0.016278 |
| GO: 0072562 | Blood microparticle | 21/1873 | 0.029202 |

Table S2. GO enrichment analysis of Molecular Function based on RNA-Seq data

| ID | Description | GeneRatio | *P* value |
| --- | --- | --- | --- |
| GO: 0019838 | Growth factor binding | 47/1815 | 2.23E-11 |
| GO: 0008201 | Heparin binding | 46/1815 | 3.79E-11 |
| GO: 0005539 | Glycosaminoglycan binding | 54/1815 | 4.85E-11 |
| GO: 1901681 | Sulfur compound binding | 59/1815 | 8.69E-11 |
| GO: 0005201 | Extracellular matrix structural constituent | 21/1815 | 9.59E-09 |
| GO: 0017147 | Wnt-protein binding | 14/1815 | 6.79E-05 |
| GO: 0005520 | Insulin-like growth factor binding | 14/1815 | 0.0001 |
| GO: 0005178 | Integrin binding | 28/1815 | 0.0001 |
| GO: 0008237 | Metallopeptidase activity | 43/1815 | 0.0001 |
| GO: 0019199 | Transmembrane receptor protein kinase activity | 23/1815 | 0.0001 |
| GO: 0030246 | Carbohydrate binding | 46/1815 | 0.0004 |
| GO: 0061134 | Peptidase regulator activity | 40/1815 | 0.0007 |
| GO: 0005126 | Cytokine receptor binding | 49/1815 | 0.0008 |
| GO: 0048407 | Platelet-derived growth factor binding | 8/1815 | 0.0009 |
| GO: 0050840 | Extracellular matrix binding | 17/1815 | 0.0009 |
| GO: 0005518 | Collagen binding | 17/1815 | 0.0046 |
| GO: 0005160 | Transforming growth factor beta receptor binding | 13/1815 | 0.03756 |
| GO: 0050431 | Transforming growth factor beta binding | 7/1815 | 0.04039 |

Table S3. GO enrichment analysis of Cellular Component based on ITRAQ data

| ID | Description | GeneRatio | *P* value |
| --- | --- | --- | --- |
| GO: 0005615 | Extracellular space | 34/66 | 2.03E-25 |
| GO: 0031012 | Extracellular matrix | 18/66 | 1.79E-23 |
| GO: 0005578 | Proteinaceous extracellular matrix | 20/66 | 8.07E-23 |
| GO: 0005581 | Collagen trimer | 9/66 | 7.73E-12 |
| GO: 0070062 | Extracellular exosome | 30/66 | 1.11E-09 |
| GO: 0005604 | Basement membrane | 5/66 | 4.07E-05 |
| GO: 0031091 | Platelet alpha granule | 3/66 | 0.0011 |
| GO: 0005584 | Collagen type I trimer | 2/66 | 0.0086 |
| GO: 0005588 | Collagen type V trimer | 2/66 | 0.0128 |
| GO: 0072562 | Blood microparticle | 3/66 | 0.0414 |
| GO: 0005605 | Basal lamina | 2/66 | 0.0422 |

| ID | Description | GeneRatio | *P* value |
| --- | --- | --- | --- |
| GO: 0005201 | Extracellular matrix structural constituent | 10/66 | 8.02E-14 |
| GO: 0008201 | Heparin binding | 11/66 | 7.79E-12 |
| GO: 0005509 | Calcium ion binding | 17/66 | 2.59E-09 |
| GO: 0050840 | Extracellular matrix binding | 5/66 | 4.00E-06 |
| GO: 0005540 | Hyaluronic acid binding | 3/66 | 0.0033 |
| GO: 0004222 | Metalloendopeptidase activity | 4/66 | 0.0126 |
| GO: 0004252 | Serine-type endopeptidase activity | 4/66 | 0.0241 |
| GO: 0043394 | Proteoglycan binding | 2/66 | 0.0332 |
| GO: 0004867 | Serine-type endopeptidase inhibitor activity | 3/66 | 0.0424 |
| GO: 0005539 | Glycosaminoglycan binding | 2/66 | 0.0425 |
| GO: 0008191 | Metalloendopeptidase inhibitor activity | 2/66 | 0.0471 |

Table S4. GO enrichment analysis of Molecular Function based on ITRAQ data
